# Plumericin inhibits proliferation of vascular smooth muscle cells by blocking STAT3 signaling via *S*-glutathionylation

**DOI:** 10.1038/srep20771

**Published:** 2016-02-09

**Authors:** Elke H Heiss, Rongxia Liu, Birgit Waltenberger, Shafaat Khan, Daniel Schachner, Paul Kollmann, Kristin Zimmermann, Muris Cabaravdic, Pavel Uhrin, Hermann Stuppner, Johannes M Breuss, Atanas G Atanasov, Verena M Dirsch

**Affiliations:** 1 Department of Pharmacognosy, University of Vienna, Althanstrasse 14, 1090 Vienna, Austria; 2 Institute of Pharmacy (Pharmacognosy) and Center for Molecular Biosciences Innsbruck (CMBI), University of Innsbruck, Innsbruck, Austria; 3 Center for Physiology and Pharmacology, Institute for Vascular Biology and Thrombosis Research, Medical University of Vienna, Vienna, Austria; 4 Department of Zoology, University of Sargodha, Sargodha, Pakistan

## Abstract

The etiology of atherosclerosis and restenosis involves aberrant inflammation and proliferation, rendering compounds with both anti-inflammatory and anti-mitogenic properties as promising candidates for combatting vascular diseases. A recent study identified the iridoid plumericin as a new scaffold inhibitor of the pro-inflammatory NF-κB pathway in endothelial cells. We here examined the impact of plumericin on the proliferation of primary vascular smooth muscle cells (VSMC). Plumericin inhibited serum-stimulated proliferation of rat VSMC. It arrested VSMC in the G1/G0-phase of the cell cycle accompanied by abrogated cyclin D1 expression and hindered Ser 807/811-phosphorylation of retinoblastoma protein. Transient depletion of glutathione by the electrophilic plumericin led to *S*-glutathionylation as well as hampered Tyr705-phosphorylation and activation of the transcription factor signal transducer and activator of transcription 3 (Stat3). Exogenous addition of glutathione markedly prevented this inhibitory effect of plumericin on Stat3. It also overcame downregulation of cyclin D1 expression and the reduction of biomass increase upon serum exposure. This study revealed an anti-proliferative property of plumericin towards VSMC which depends on plumericin’s thiol reactivity and *S*-glutathionylation of Stat3. Hence, plumericin, by targeting at least two culprits of vascular dysfunction –inflammation and smooth muscle cell proliferation -might become a promising electrophilic lead compound for vascular disease therapy.

Atherosclerosis is the underlying cause of the majority of cardiovascular diseases, which represent the leading cause of mortality worldwide^1^,^2^ The pathology of atherosclerosis is from its early stages driven by a chronic ongoing inflammatory state causing leukocyte recruitment to the vessel wall, induced proliferation of vascular smooth muscle cells (VSMC) and generation of a collagen-rich matrix. These result in the formation of plaques in the intima, the inner-most layer of large and mid-sized arteries, causing their narrowing^3^,^4^,^5^. Surgical interventions that aimed to repair the obstructed vessel lumen, such as angioplasty, stenting, and bypass, frequently fail due to VSMC-driven restenosis, which is the most feared complication of these therapeutic procedures^6^. Since inflammation and increased proliferation of VSMCs are two major hallmarks of plaque formation and restenosis^7^,^8^, the identification of compounds inhibiting both, inflammation and VSMC proliferation, could be of therapeutic relevance for combating vascular disease.

Medicinal plants have been used for millennia for the treatment of diseases, and now still provide an effective source for new drug discovery^9^,^10^,^11^. Aiming at the identification of new natural products with anti-inflammatory potential, we have previously investigated components contained in the bark material of the Amazonian tree *Himatanthus sucuuba* (Spruce ex Müll.Arg.) Woodson (Apocynaceae)^12^,^13^. In folk medicine, its bark and latex are used as anti-inflammatory, antitumor, analgesic, and antiulcer agents^14^,^15^,^16^. Employing bioactivity-guided isolation we identified the spirolactone iridoid plumericin as a major bioactive constituent of *H. sucuuba*. This compound had been shown to exhibit antiparasitic^17^, antimicrobial^18^, and antifungal^19^ activities. Our former work additionally characterized plumericin as a potent inhibitor of the NF-κB pathway in the vasculature with anti-inflammatory activity *in vitro* and *in vivo*^13^.

Aim of the present study was to examine whether plumericin might target another culprit of vascular disease besides the inflammation. Specifically, we investigated the influence of plumericin on VSMC proliferation *in vitro* and the underlying molecular mode of action.

## Results

### Plumericin inhibits proliferation of vascular smooth muscle cells (VSMC)

**Figure 1:**
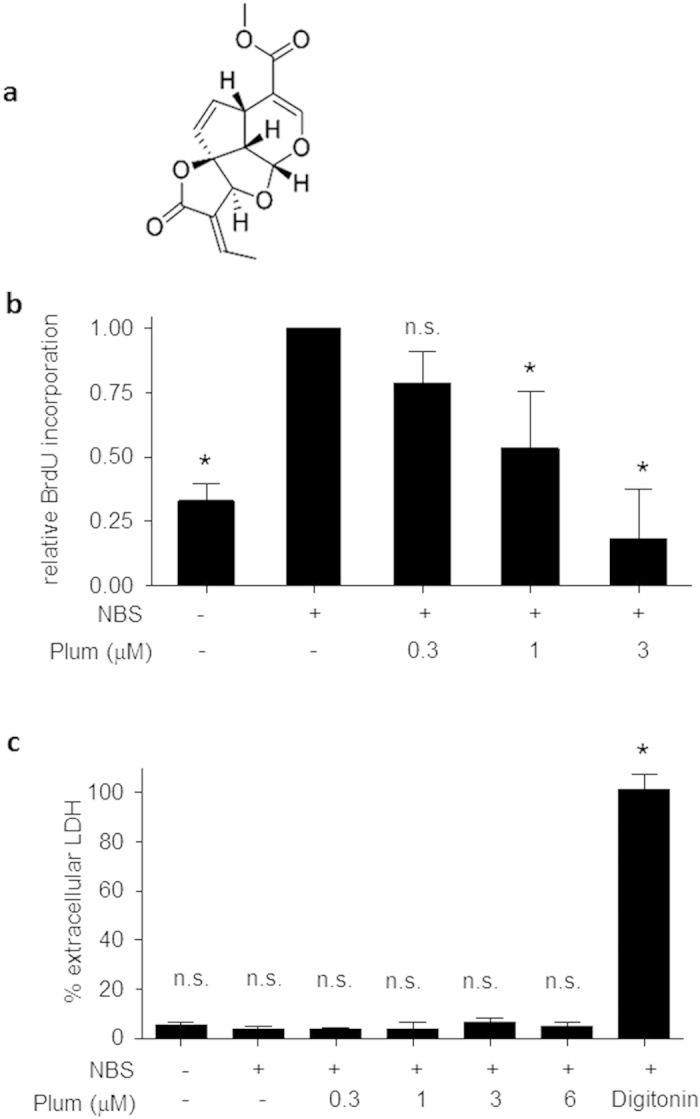
Plumericin inhibits serum-induced VSMC proliferation (**a**) Chemical structure of plumericin (**b**) Quiescent VSMC were treated as indicated for 30 min and then stimulated with serum (10% NBS) for 24 h, and cell proliferation was quantified by assessing BrdU incorporation into newly synthesized DNA (mean + SD, n = 3, *p < 0.05 (vs serum-stimulated cells); ANOVA, Bonferroni) (**c**) Quiescent VSMC were treated as indicated and stimulated with serum (NBS, 10%) for 48 h. Then cell membrane integrity was assessed by quantification of extracellular lactate dehydrogenase (LDH) as described in the methods section. Digitonin (100 μg/mL) was used as a positive cytotoxic control. (mean + SD, n = 3, *p < 0.05 (vs serum-stimulated cells), ANOVA, Bonferroni).

The complex etiology of vessel lumen reduction includes inflammatory processes and aberrant cell proliferation. This study investigated whether plumericin (Fig. 1a), already identified as inhibitor of the NF-κB signaling pathway in endothelial cells^13^, also interferes with the proliferation of vascular smooth muscle cells (VSMC) and could, thus, be further developed into a potential dual defense against (re)stenosis. Indeed, plumericin concentration-dependently (IC_50_ value of 1.11 μM) inhibited serum-induced incorporation of the thymidine analogue 5-bromo-2′-deoxy-uridin (BrdU) in VSMC, which is indicative of impaired DNA replication during cell division (Fig. 1b). Inhibition of cell proliferation was corroborated by measuring cellular metabolic activity (resazurin conversion, IC_50_ value of 1.08 μM) and by biomass staining (crystal violet, IC_50_ value of 2.93 μM) in response to plumericin (Supplemental Fig. 1a,b). For comparison, the IC_50_ value of taxol, a known inhibitor of VSMC proliferation and used as stent coating agent^20^, ranged between 180 and 230 nM in those assays (data not shown). The observed reduction in proliferation was not due to apparent cytotoxicity and membrane disintegration as VSMC did not release cytosolic lactate dehydrogenase (LDH) upon treatment with the used concentrations of plumericin for 48 hours (Fig. 1c). Moreover, treated cells did not display any morphological changes or detachment indicative for apoptosis or cell death (Supplemental Fig. 1 c).

### Plumericin arrests VSMC in the G1/G0 phase of the cell cycle and blocks cyclin D1 upregulation

**Figure 2:**
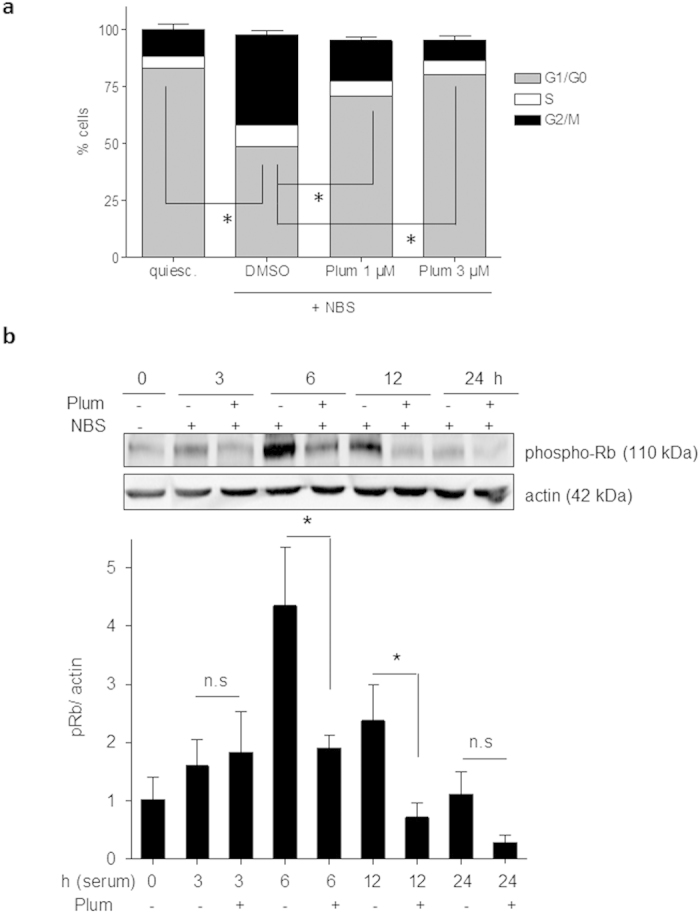
Plumericin arrests VSMC in G0/G1 of the cell cycle. (**a**) Serum-deprived quiescent (quiesc.) VSMC were pretreated with solvent (DMSO) or plumericin as indicated for 30 min and then exposed to serum (10% NBS) for 16 h before cell nuclei were stained with PI and analyzed by flow cytometry. Compiled evaluation of three independent experiments is shown (mean +SD; *p < 0.05 (compared groups (% cells in G1 phase) are indicated by lines), ANOVA, Bonferroni). (**b**) Quiescent VSMC were pretreated with 3 μM plumericin (Plum) for 30 min, exposed to serum (10% NBS) for the indicated periods of time and then lysed. Total cell lysates were subjected to western blot analysis for phospho Rb (Ser 807/811) and actin as loading control. Representative cropped blots (for raw data see Supplemental Fig. 9) and compiled densitometric data from three independent experiments are depicted (n = 3, *p < 0.05 (compared groups are indicated by lines), ANOVA, Dunnett).

**Figure 3:**
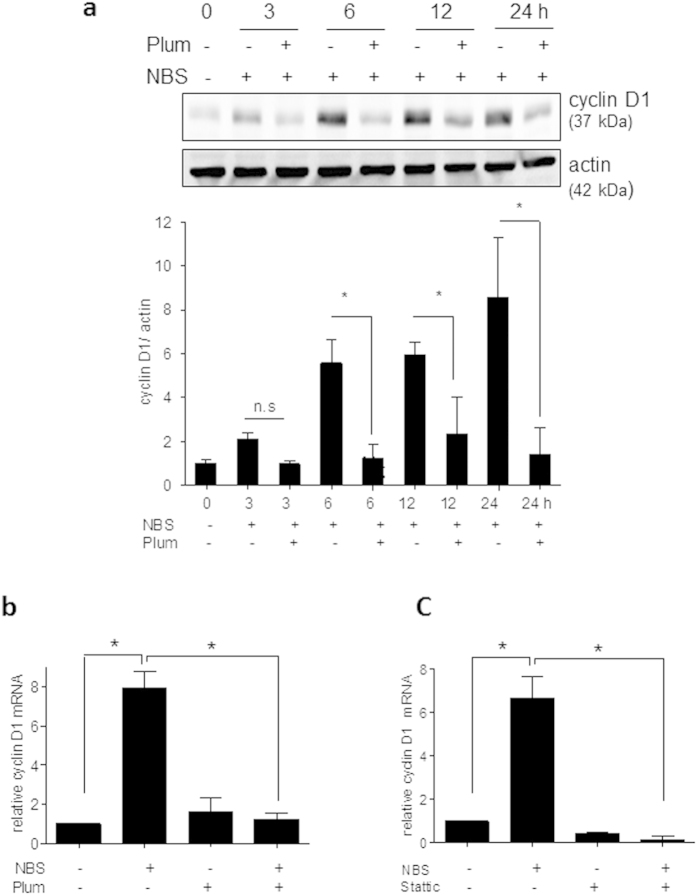
**Plumericin and Stattic inhibit serum-induced cyclin D1 upregulation in VSMC**. (**a**) Quiescent VSMC were pretreated with 3 μM plumericin (Plum) for 30 min and then exposed to serum (10% NBS) as indicated. After lysis, total cell lysates were subjected to immunoblot analysis for cyclin D1 and actin. Representative cropped blots (for raw data see Supplemental Fig. 9) as well as compiled densitometric data from three independent experiments are depicted (n = 3, *p < 0.05 (compared groups are indicated by lines), ANOVA Bonferroni). Quiescent VSMC were pretreated with plumericin (3 μM, Plum) (**b**) or Stattic (10 μM) (**c**) for 30 min prior to exposure to serum (10% NBS) for 6 h. RNA was extracted, transcribed to cDNA, subjected to qPCR analysis for cyclin D1 as target gene or HRPT-1 and 18S rRNA as reference genes. The graphs depict compiled data (2^−ΔΔCt^) from three independent experiments each. (*p < 0.05; (compared groups are indicated by lines), ANOVA, Bonferroni).

Intrigued by the anti-proliferative activity of plumericin in the phenotypic assays we took a closer look at its molecular mode of action in VSMC. Cell cycle progression in serum-stimulated VSMC was analyzed by flow cytometry. Here, vehicle-treated and serum-deprived cells were arrested in the G1/G0 phase of the cell cycle (Supplemental Fig. 2a), which they left for S and G2/M phase after incubation (16 h) with growth factors present in serum (Supplemental Fig. 2b). In contrast, plumericin-treated cells (1 and 3 μM) were retained in the G0/G1 phase of the cell cycle despite growth factor stimulation (Supplemental Fig. 2c,d). The significant arrest in G0/G1 phase of the cell cycle induced by plumericin (Fig. 2a) was consistent with an observed hypophosphorylation of retinoblastoma protein (Rb) at Ser 807/811 over time (Fig. 2b), known to hamper the transition to S phase^21^,^22^,^23^. Notably, the identified plumericin-mediated arrest in G0/G1 was also in accordance with the observed reduction of BrdU incorporation occurring in S phase of the cell cycle (Fig. 1b). The progression through the different cell cycle phases is regulated by the dynamic expression of phase-specific cyclins and the subsequent activation of corresponding cyclin- dependent kinases (CDK). The G1→S transition highly relies on Rb phosphorylation by cyclin D1-CDK 4/6 complexes^21^,^22^,^23^. Western blot analyses revealed that plumericin (3 μM) abrogated the time-dependent expression of cyclin D1, which was seen in vehicle-treated cells as early as 3 h after cell exposure to serum (Fig. 3a). QPCR analyses revealed a complete inhibition of serum-induced cyclin D1 mRNA upregulation in the presence of plumericin (Fig. 3b) suggesting activity of plumericin at the transcriptional or posttranscriptional level.

### Cyclin D1 upregulation is Stat3- dependent in serum-stimulated VSMC

Expression of cyclin D1 is controlled by cues from multiple signaling pathways and transcription factors, including mitogenic kinases, NF-κB or signal transducer and activator of transcription (Stat) 3^24^. One of the earliest events downstream of growth factor stimulation is activation of mitogen-activated protein kinases (MAPK), i.e. ERK1/2, JNK and p38, and Akt/PKB. Plumericin (3 μM) did not interfere with serum-induced phosphorylation of ERK1/2 at Thr202/Tyr204 or AKT at Ser473 (Supplemental Fig. 3a) and enhanced the phosphorylation of p38 at Thr180/Tyr182 and JNK at Thr183 /Tyr185 (Supplemental Fig. 3b,c). The latter effect could not be causally linked with the anti-proliferative action of plumericin as pharmacological inhibitors of p38 or JNK failed to overcome the plumericin-mediated growth arrest (Supplemental Fig. 4a,b). The inhibition of NF-κB activation by plumericin in endothelial and human embryonal kidney cells by impaired IκB degradation^13^ together with the reported role of NF-κB for cyclin D1 regulation rendered NF-κB another conceivable target responsible for our observations. However, neither serum nor plumericin obviously affected IκB levels (Supplemental Fig. 5a,b), consistent with previous findings showing that NF-κB apparently does not play a major role in proliferating VSMC^25^. An influence of non-canonical, IκB-independent NF-κB signaling on cyclin D1 expression cannot be excluded in the used VSMC model, though. Employing the known selective Stat3 inhibitor Stattic^26^, which was confirmed to successfully inhibit serum-induced Stat3 phosphorylation under our experimental conditions (data not shown), we identified Stat3 as important factor in the serum induced induction of cyclin D1 (Fig. 3c).

### Plumericin interferes with Stat3-dependent mitogenic signaling by causing transient GSH depletion

**Figure 4:**
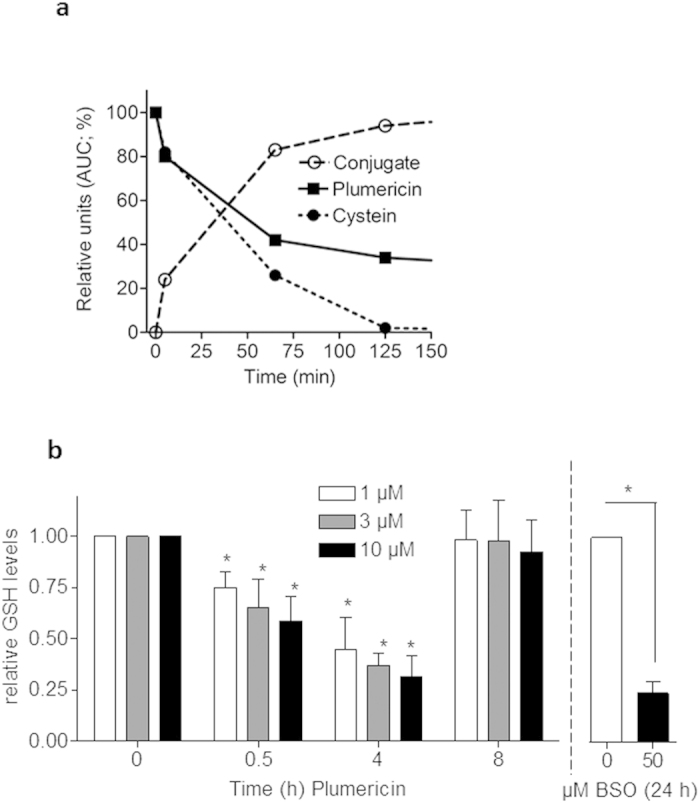
Plumericin forms adducts with cysteine and transiently depletes VSMC from GSH. (**a**) Plumericin and cysteine methyl ester were co-incubated as described in the Methods sections. After the indicated period of time the amount of free plumericin, free cysteine methyl ester and of the respective adduct were determined by HPLC. Values are given as percentages of the maximum areas under the curves (AUCs) (**b**) Quiescent VSMC were treated with different concentrations of plumericin for the indicated period of time or BSO (50 μM, 24 h) before the intracellular level of free GSH was determined as described in the Methods section. Bar graph depicts compiled data from three independent experiments expressed as fold untreated control. (*p < 0.05 (vs t_o_ of the respective plumericin concentration or indicated by lines); ANOVA, Dunnett)

Stat3 is a transcription factor highly susceptible to redox regulation and thiol modification^27^,^28^,^29^. Accordingly, Stat3 was found to be glutathionylated at Cys328 and Cys542 under conditions of glutathione (GSH) depletion by redox or electrophilic stress and hereby prevented from being phosphorylated and activated^30^,^31^,^32^. The chemical structure of plumericin with an exocyclic α,β-unsaturated carbonyl group suggests high thiol reactivity, which could be confirmed *in vitro* where plumericin readily formed adducts with free cysteine (Fig. 4a). Plumericin also depleted VSMC transiently from GSH, the most abundant cellular thiol for detoxification of ROS or xenobiotics. In the presence of 1-10 μM plumericin, intracellular GSH levels dropped concentration-dependently between 30 min and 4 h and then returned to basal levels (=approximately 14 μmol/g protein in the used cells) after 8 h (Fig. 4b). Incubation with 50 μM buthionine sulfoximine (BSO) for 24 h led to an expected drop of cellular GSH.

**Figure 5:**
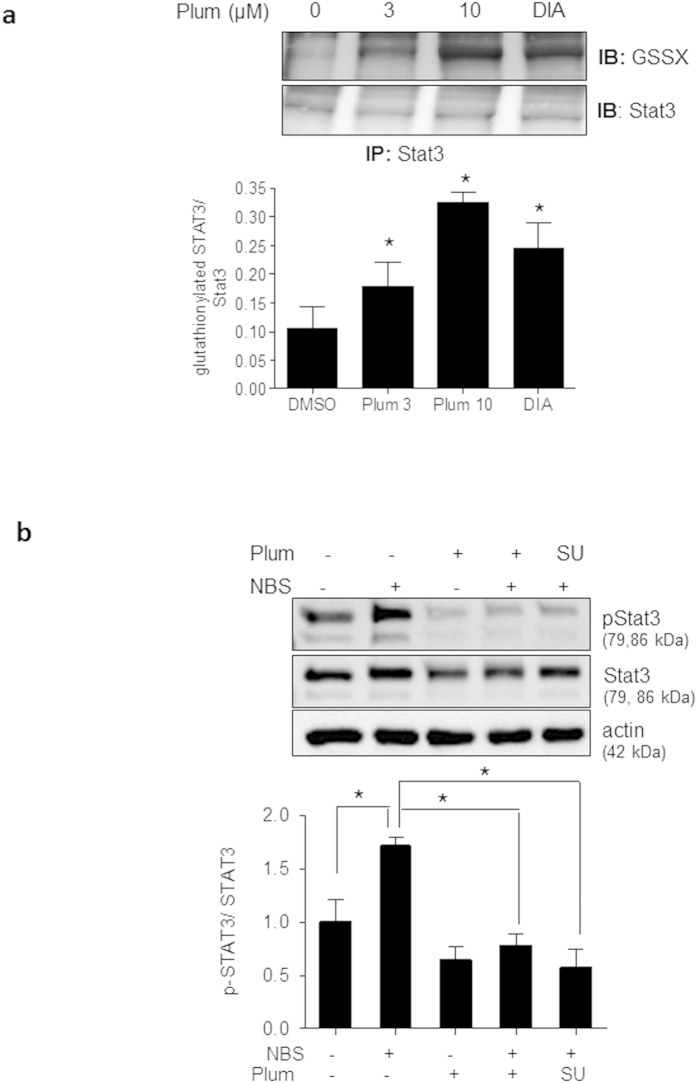
**Plumericin leads to glutathionylation of Stat3 and inhibits serum-induced phosphorylation of Tyr705 of Stat3**. (**a**) Quiescent VSMC were treated with vehicle (DMSO), plumericin (Plum, 3 and 10 μM) or diamide (DIA, 1 mM) for 30 min before total cell lysates were subjected to immunoprecipitation for Stat3 and immunoblotting for protein-linked GSH (GSSX) and Stat3. Representative blots as well as compiled densitometric evaluations are depicted. (n = 3, *p < 0.05 (vs DMSO control); ANOVA, Dunnett). (**b**) Quiescent VSMC were pretreated with vehicle (DMSO), 3 μM plumericin (Plum) or 5 μM SU6656 (SU, Src inhibitor) for 30 min prior to exposure to serum (10% NBS) for 10 min. Total cell lysates were subjected to immunoblot analysis for phospho-Stat3 (Tyr705), Stat3 and actin. Compiled densitometric data from three independent experiments are shown in the bar graph (n = 3, *p < 0.05 (compared groups are indicated by lines), ANOVA, Dunnett). Shown blots are cropped (for larger images please see Supplemental Fig. 9).

**Figure 6:**
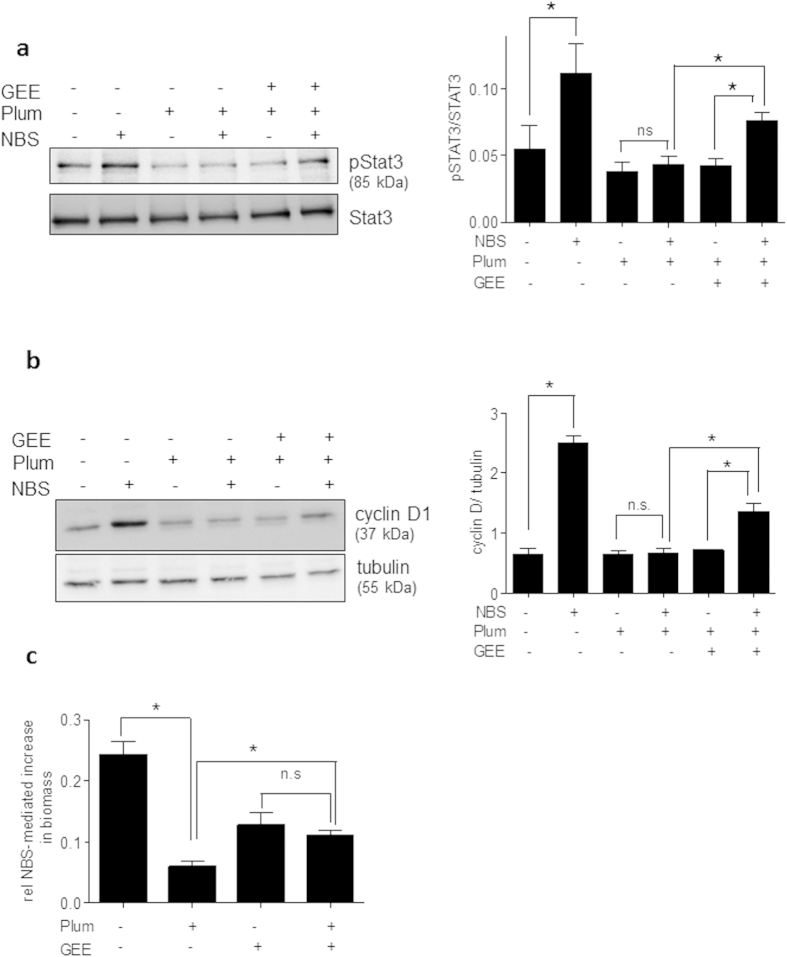
Exogenous addition of GSH, in form of cell permeable ethyl ester (GEE), hinders plumericin to inhibit Stat3 phosphorylation, cyclin D1 upregulation and proliferation in VSMC. Quiescent VSMC were treated with 2 mM GEE for 18 h and thoroughly washed. Cells were then treated with plumericin (Plum, 3 μM) for 30 min and stimulated with serum (10% NBS) for 10 min, 8 h or 48 h before subjected to western blot analysis for (**a**) phospho Stat3 (Tyr705), Stat3, and actin, or (**b**) cyclin D1 and tubulin or to (**c**) biomass stain by crystal violet. Depicted blots are cropped (for raw data see Supplemental Fig. 9) and representative for three independent experiments. Bar graphs depict compiled data from the three independent experiments (*p < 0.05 (compared groups are indicated by lines), ANOVA, Bonferroni).

As just mentioned, GSH depletion has been shown to trigger *S*-glutathionylation of proteins, including Stat3^30^,^33^,^34^,^35^. Based on the observed pivotal role of Stat3 for cyclin D1 expression and the transient GSH depletion by plumericin it appeared intriguing to hypothesize that *S*-glutathionylation and subsequent inhibition of Stat3^30^,^31^ account for the reduced cyclin D1 expression in the presence of plumericin. To test this hypothesis, immunoprecipitation of endogenous Stat3 from VSMC that had been treated with vehicle, plumericin (3 and 10 μM) or diamide (1 mM; positive control) for 30 min and subsequent immunoblotting for protein-attached GSH were performed. Obtained results showed that plumericin was indeed able to cause a significant covalent attachment of GSH to Stat3 (Fig. 5a). In line with reported impaired activation of Stat3 upon *S*-glutathionylation^30^,^31^ plumericin prevented serum-induced Stat3 phosphorylation at Tyr705, which represents an essential step for Stat3 dimerization, nuclear translocation and transcriptional activity. The Src inhibitor SU6656 was used as positive control for known inhibition of Stat3 phosphorylation^36^(Fig. 5b). Furthermore, administration of exogenous GSH in form of its cell-permeable ethyl ester (GEE) significantly diminished the plumericin-mediated inhibition of Stat3 tyrosine phosphorylation and of cyclin D1 upregulation. (Fig. 6a,b). GEE also significantly abrogated the inhibitory effect of plumericin on serum-induced increase in cell mass (Fig. 6c). Compared to control cells, GEE-treated VSMC showed a weaker proliferative response to serum exposure. This is most likely due to a disturbed ROS-based signal transduction which is crucial for the initial phase after mitogenic stimulation. A comparable picture arose when performing cell cycle analysis in the presence of GEE: Addition of serum made quiescent VSMC move to S-phase, which was markedly inhibited by plumericin. Co-treatment with GEE reduced the number of cells moving into S-phase after serum exposure, but also the effect of plumericin on the serum-induced cell cycle progression (Supplemental Fig. 6). Overall, GSH depletion by plumericin could be confirmed to contribute to the reduced STAT3 phosphorylation, cyclin D1 expression and proliferation in serum-stimulated VSMC.

## Discussion

This study demonstrates that plumericin potently inhibits serum-induced proliferation of VSMCs with an IC_50_ value in the low micromolar range. Transient depletion of cells from intracellular GSH upon plumericin treatment, *S*-glutathionylation and impaired activation of Stat3, as well as abrogated cyclin D1 expression are important features of the hampered cell cycle progression upon serum stimulation.

Plumericin stalled VSMC in the G0/G1 phase of the cell cycle which was accompanied by a reduction in cyclin D1 expression and subsequent CDK-dependent Rb phosphorylation. In line with previous studies^37^,^38^,^39^ Stat3 emerged as crucial mitogenic signaling molecule for VSMC and as a promising, though possibly still underestimated drug target in the field of vasculoproliferative disease research^40^. Based on the findings with the selective Stat3 inhibitor Stattic, this transcription factor appeared as the main mediator for cyclin D1 upregulation in our model system. Plumericin hindered serum-induced Stat3 phosphorylation and activation by transiently depleting cells from intracellular GSH and by causing *S-*glutathionylation of Stat3 (most likely at Cys328 and Cys542) which interferes with Tyr705 phosphorylation^30^,^31^. Interestingly, plumericin shares the structural feature of an α,β-unsaturated carbonyl group acting as electrophilic Michael system with cynaropicrin, dehydrocustuslactone and costulonide, which have also been reported to deplete cells from GSH and to trigger *S*-glutathionylation of Stat3, although at remarkably higher concentrations (25–50 μM) in THP-1 cells^41^,^32^. The thiol reactivity of plumericin also underlies the previously discovered anti-inflammatory action of plumericin in endothelial cells^13^. As both endothelial inflammation and VSMC proliferation promote vascular pathologies such as arteriosclerosis or restenosis, plumericin occurs as a promising multi target hit compound for further development to a novel vasoprotective lead. Of note, a first small scale *in vivo* study using the murine femoral cuff model showed significantly reduced neointima formation in plumericin-treated arteries (Supplemental Fig. 7a). Furthermore and in contrast to taxol, plumericin did not heavily affect endothelial cell viability in concentrations inhibiting VSMC growth, an asset for potential anti-restenotic agents (Supplemental Fig. 7b). Future *in vivo* studies are warranted to corroborate and further analyze this promising activity profile.

Addition of exogenous GSH could significantly attenuate but not fully overcome the Stat3-inhibitory and antiproliferative activity of plumericin suggesting involvement of additional GSH-independent mechanisms. Based on the unaltered phosphorylation of Src at Y418, plumericin did not appear as Src inhibitor (data not shown). Plumericin did not show marked toxicity in the tested concentration range ruling out an aberrant random and harmful covalent modification of cellular cysteine residues. Electrophilic compounds are known to lead to activation of the transcription factor nuclear factor-erythroid 2 p45-related factor 2 (Nrf2), which launches a cellular detoxification response protecting the cells against xenobiotic and oxidative stress^42^. Accordingly, plumericin was able to concentration-dependently activate Nrf2 in a luciferase reporter gene assay and trigger expression of heme oxygenase 1 (HO-1), a Nrf2 target gene, in VSMC (Supplemental Fig. 8a,b). Activation of Nrf2 most likely also accounts for the restoration of cellular GSH levels after initial depletion by plumericin, as the key enzymes of GSH biosynthesis are Nrf2′s target genes^43^. Although conceivable from previous reports^44^,^45^,^46^ the induced Nrf2/HO1 axis could not be causally connected to the antiproliferative activity in VSMC (Supplemental Fig. 8c). Nonetheless the Nrf2/HO-1 axis may add further positive properties to the vasoprotective pharmacological profile of plumericin. Likewise, several studies showed a favorable influence of HO-1 and/or Nrf2 on endothelial function, monocyte adhesion, migration of VSMC, cholesterol homeostasis and redox stress (e.g^47^,^48^,^49^,^50^,^51^.). Whether plumericin affects those processes needs to be further investigated. Notably, plumericin shares its concomitant influence on the Nrf2-, NF-κB- and Stat3 transcription factor pathways with several other electrophilic compounds/drugs of high pharma/nutraceutical interest, including the synthetic triterpenoid CDDO-IM^52^,^53^,^54^, dimethylfumarate^55^,^56^,^57^ and the turmeric-derived curcumin^58^,^59^,^60^. Thus, it seems that those three pathways are heavily susceptible to thiol modification and their modulation correlates with a desirable bioactivity profile^61^. It remains to be seen whether the positive influence of plumericin can also be extended to the field of cancer or neurodegenerative diseases as in the case of the mentioned electrophilic “blockbusters”. Indeed, a recent study already reported successful growth inhibition of two immortalized leukemic cell lines by plumericin^62^.

To conclude, we uncover growth inhibition of primary VSMC by plumericin in the low micromolar range and link this observation with *S*-glutathionylation and inhibition of Stat3 and with subsequently impaired mitogenic signaling. This study complements the known bioactivity profile of plumericin, corroborates Stat3 as promising drug target in vascular pathologies and stresses the potential of thiol reactive compounds as polypharmacological agents with an overall favorable activity profile^63^.

## Materials and Methods

### Chemicals and reagents

Plumericin was isolated and characterized from the powdered bark of *Himatanthus sucuuba* as previously described in detail^12^. The Src inhibitor SU6656 was obtained from Merck Millipore (Vienna, Austria) and the sepharose A/G beads came from Santa Cruz (Heidelberg, Germany). The chemoluminescent 5-bromo-2′-deoxyuridine (BrdU) cell proliferation kit was obtained from Roche Diagnostics (Vienna, Austria). All other used reagents and chemicals were of analytical grade and bought from Sigma-Aldrich (Vienna, Austria). Antibodies against phospho-Stat3 (Tyr705) (#9131), phospho-retinoblastoma protein (Ser807/811) (#9308), as well as Stat3 (#9132), cyclin D1 (DCS6, #2926) and the secondary horseradish-peroxidase-coupled antibodies were purchased from New England BioLabs (Frankfurt, Germany). The anti-actin antibody was bought from MP Biomedicals (Eschwege, Germany) and the anti-GSSX antibody from Virogen (Watertown, MA, USA). Primers for qPCR were designed with the Primer 3 software and obtained from Invitrogen (Carlsbad, CA, USA). RNA isolation PeqGOLD Total RNA kit and Reverse Tanscription kit came from Peqlab (Erlangen, Germany) and Applied Biosystems (vie LifeTech, Vienna, Austria), respectively. The Light CyclerTM LC480 SYBR Green I Master reagent was obtained from Roche Diagnostics (Vienna, Austria).

### Cell culture

Primary rat aortic VSMC were purchased from Lonza (Braine-L’Alleud, Belgium). They were cultivated in Dulbecco’s modified essential medium (DMEM)–F12 (1:1) supplemented with 20% serum, 30 μg/mL gentamicin, and 15 ng/mL amphotericin B (Braine-L’Alleud, Belgium) and used between passage 7 and 15 (proliferative/synthetic phenotype).

### Assessment of cell proliferation via 5-bromo-2′-deoxyuridine (BrdU) incorporation

VSMC were seeded at a density of 5 × 10^3 ^cells/well in 96-well plates. After 24 h, cells were serum-deprived for 24 h to render them quiescent. Quiescent cells were pretreated for 30 min with plumericin or vehicle (0.1% DMSO) as indicated and subsequently stimulated with newborn bovine serum (NBS; 10% final concentration). BrdU was added 2 h after NBS stimulation, and after further 22 h the *de novo* DNA synthesis was estimated by a cell proliferation ELISA kit according to the manufacturer´s instructions.

### Assessment of cytotoxicity via release of lactate dehydrogenase (LDH)

VSMC were seeded at a density of 5 × 10^3 ^cells/well in 96-well plates. After 24 h, cells were serum-deprived for another 24 h to render them quiescent. Cells were pretreated for 30 min with either plumericin, the positive control digitonin (100 μg/mL), or vehicle (0.1% DMSO) as indicated, and subsequently stimulated for 24 h with 10% NBS. Quantification of the release of the soluble cytosolic protein lactate dehydrogenase (LDH) can be utilized to evaluate the loss of cell membrane integrity that is associated with cell death. For this, the supernatant of the treated cells was measured for LDH activity. For determination of the total LDH activity, identically treated samples were incubated for 45  min with 1% Triton X-100. The released and total LDH enzyme activity was determined for 30 min in the dark in the presence of 4.5  mg/mL lactate, 0.56  mg/mL NAD^+^, 1.69  U/mL diaphorase, 0.004% (w/v) BSA, 0.15% (w/v) sucrose, and 0.5  mM 2-p-iodophenyl-3-nitrophenyl tetrazolium chloride (INT). The absorbance was measured at 490 nm after the enzyme reaction was quenched with 1.78  mg/mL oxymate. Percentage of extracellular LDH enzyme activity was calculated to evaluate the potential effects on cell viability.

### Cell cycle analysis via flow cytometry

VSMC were seeded at a density of 1 × 10^4 ^cells/well in 12-well plates. After 24 h, the cells were serum-deprived for 24 h to render them quiescent. Cells were pretreated for 30 min with plumericin (1 or 3 μM) or vehicle (0.1% DMSO) as indicated, and subsequently stimulated for 16 h with 10% NBS. Then, the cells were trypsinized, washed once with PBS, and resuspended in a hypotonic propidium iodide (PI) solution containing Triton X-100 (0.1% v/v), sodium citrate (0.1% w/v), and PI (50 μg/mL). After overnight incubation at 4 °C, PI-stained nuclei were analyzed by flow cytometry (excitation 488 nm, emission 585 nm; FACScalibur; BD Biosciences, Germany).

### Protein extraction, SDS PAGE and immunoblot analysis

VSMC were seeded at a density of 3 × 10^5 ^cells/well in 6-well plates. After 24 h, cells were serum-deprived for another 24 h and treated as indicated. Then cells were lysed followed by sonication before 15–20 μg protein were subjected to SDS-PAGE electrophoresis and immunoblot analysis as previously described in detail^64^,^39^,^44^. All antibodies were diluted following the recommendation of the providing company. Proteins were visualized using enhanced chemoluminescence and a LAS-3000 luminescent image analyzer (Fujifilm) with AIDA software (Raytest) for densitometric evaluation.

### Real time quantitative polymerase chain reaction (RT-qPCR)

RNA isolation and subsequent cDNA synthesis were performed according to the instructions of the respective kit manufacturer. The real-time SybrGreen-based quantitative PCR was carried out in a reaction volume of 15 μL. Forward and reverse primers for rat cyclin D1 as target gene (fwd: GCA GCG GTA GGG ATG AAA TA; rev: GGA ATG GTT TTG GAA CAT GG) as well as 18S rRNA (fwd: GAA TTG ACG GAA GGG CAC CAC CAG; rev: GTG CAG CCC CGG ACA TCT AAG G) and rat hypoxanthinphosphoribosyltransferase (HPRT1) (fwd: AAG CTT GCT GGT GAA AAG GA; rev: ATT CAA CTT GCC GCT GTC TT) as reference genes were obtained from Invitrogen (Carlsbad, CA, USA). PCR contained one denaturation step (5 min at 95 °C) and up to 55 amplification cycles (denaturation: 10 sec at 95 °C, annealing 20 sec at 55 °C and elongation 30 sec at 72 °C). Melting curves of the amplified DNA were analyzed to make sure that the PCR resulted in amplification of one specific product only, which was reconfirmed by a single distinct band on an agarose gel. Data were analyzed using Light Cycler® LC480 Software (Roche Diagnostics, Vienna, Austria) and the 2^−∆∆Ct^ method.

### Immunoprecipitation of Signal transducer and activator of transcription (Stat) 3

VSMC were grown in 10 cm^2^ cell culture dishes, treated as indicated and lysed (under non reducing conditions). Anti-Stat3 antibody (1:100 dilution) was added to 750 μg protein extract and incubated on ice for 3 h. After another 45 min rolling end-over-end with protein sepharose A/G beads at 4 °C the immunoprecipitates were collected, washed three times with cold lysis buffer, separated from the beads by boiling in SDS sample buffer (without reducing agent) for 5 min and subjected to immunoblot analyses as indicated. Specificity of the Stat3 pulldown was assured in pilot experiments using an isotype control antibody.

### Determination of intracellular glutathione (GSH) levels

VSMC were seeded in 96-well plates and serum starved. Plumericin was added at the indicated concentrations for the indicated periods of time. Buthionine sulfoximine (BSO), an irreversible inhibitor γ-glutamyl cysteine ligase, was used as a positive control. Then medium was aspirated and cells were washed with PBS before subjected to three freeze thaw cycles. GSH was measured by an enzymatic recycling procedure developed for the sensitive determination of total GSH levels in cells cultured in 96-well plates. Hereby GSH is sequentially oxidized by 5,5′-dithiobis-(2-nitrobenzoic acid)(Ellman’s reagent) and reduced by NADPH in the presence of glutathione reductase. The standard protocol^65^ was coupled to a NADPH-generating system consisting of glucose 6-phosphate/glucose-6-phosphate dehydrogenase, essentially as described in^66^,^67^. GSH levels were finally normalized to the protein content of the well.

### Determination of cysteine-plumericin adducts

Plumericin (10 mM solution in acetonitrile) was added to an equal volume of L-cysteine methyl ester (9 mM in water), mixed thoroughly and incubated at room temperature. The progress of the reaction was monitored by high performance liquid chromatography (conditions: instrument: Agilent 1100 Series; stationary phase: Phenomenex Gemini® 5μm C18 110 Å (150 × 3mm); mobile phase: water (A) and acetonitrile (B); gradient: 0 min: 5% B, 30 min: 98% B; flow rate: 0.5 mL/min; temperature: 35 °C; injection volume: 10 μL). Measurements were conducted at the indicated time points, and the concentrations of plumericin, L-cysteine methyl ester and the conjugate of the two compounds were quantified by calculating the areas under the curves (AUCs) of the compound peaks at 215 nm (L-cysteine methyl ester, retention time (RT) 1.4 min and conjugate, RT 9.0 min) and 265 nm (plumericin, RT 16.0 min), respectively. Methyl ester of L-cysteine was used in this experiment in order to ensure that the reaction of plumericin takes place only with the sulfhydryl group of cysteine.

### Statistical analysis

Statistical analysis was performed using GraphPad PRISM software, version 4.03. Statistical differences among the treatment groups were analyzed by ANOVA/Bonferroni test or by Student´s *t* test (when comparing just two experimental groups). Data in the Figures represent means +SD, and the number of experiments is given in the figure legends. *P*-values <0.05 were considered significant.

## Additional Information

**How to cite this article**: Heiss, E. H. *et al.* Plumericin inhibits proliferation of vascular smooth muscle cells by blocking STAT3 signaling via S-glutathionylation. *Sci. Rep.* **6**, 20771; doi: 10.1038/srep20771 (2016).

## Supplementary Material

Supplementary Information

## References

[b1] Lusis A. J. Atherosclerosis. Nature 407, 233–241, doi: 10.1038/35025203 (2000). 11001066 PMC2826222

[b2] Legein B., Temmerman L., Biessen E. A. & Lutgens E. Inflammation and immune system interactions in atherosclerosis. Cell Mol Life Sci 70, 3847–3869, doi: 10.1007/s00018-013-1289-1 (2013). 23430000 PMC11113412

[b3] Hansson G. K. Inflammation, atherosclerosis, and coronary artery disease. N Engl J Med 352, 1685–1695, doi: 10.1056/NEJMra043430 (2005). 15843671

[b4] Libby P. Inflammation in atherosclerosis. Arterioscler Thromb Vasc Biol 32, 2045–2051, doi: 10.1161/ATVBAHA.108.179705 (2012). 22895665 PMC3422754

[b5] Libby P. Inflammation in atherosclerosis. Nature 420, 868–874, doi: 10.1038/nature01323 (2002). 12490960

[b6] Mills B., Robb T. & Larson D. F. Intimal hyperplasia: slow but deadly. Perfusion 27, 520–528, doi: 10.1177/0267659112452316 (2012). 22751382

[b7] Thyberg J., Hedin U., Sjolund M., Palmberg L. & Bottger B. A. Regulation of differentiated properties and proliferation of arterial smooth muscle cells. Arteriosclerosis 10, 966–990 (1990). 2244864 10.1161/01.atv.10.6.966

[b8] Braun-Dullaeus R. C., Mann M. J. & Dzau V. J. Cell cycle progression: new therapeutic target for vascular proliferative disease. Circulation 98, 82–89 (1998). 9665064 10.1161/01.cir.98.1.82

[b9] Kinghorn A. D., Pan L., Fletcher J. N. & Chai H. The relevance of higher plants in lead compound discovery programs. J Nat Prod 74, 1539–1555, doi: 10.1021/np200391c (2011). 21650152 PMC3158731

[b10] Cragg G. M. & Newman D. J. Natural products: a continuing source of novel drug leads. Biochim Biophys Acta 1830, 3670-3695, doi: 10.1016/j.bbagen.2013.02.008 (2013). 23428572 PMC3672862

[b11] Atanasov A. G. *et al.* Discovery and resupply of pharmacologically active plant-derived natural products: A review. Biotechnol Adv, doi: 10.1016/j.biotechadv.2015.08.001 (2015). PMC4748402 26281720

[b12] Waltenberger B., Rollinger J. M., Griesser U. J., Stuppner H. & Gelbrich T. Plumeridoid C from the Amazonian traditional medicinal plant Himatanthus sucuuba. Acta Crystallogr C 67, o409–412, doi: 10.1107/S0108270111035761 (2011). 21979978 PMC3845386

[b13] Fakhrudin N. *et al.* Identification of plumericin as a potent new inhibitor of the NF-kappaB pathway with anti-inflammatory activity *in vitro* and *in vivo*. Br J Pharmacol 171, 1676–1686, doi: 10.1111/bph.12558 (2014). 24329519 PMC3966748

[b14] Perdue G. P. & Blomster R. N. South American plants III: Isolation of fulvoplumierin from Himatanthus sucuuba (M. Arg.) Woodson (Apocynaceae). J Pharm Sci 67, 1322–1323 (1978). 690844 10.1002/jps.2600670936

[b15] de Miranda A. L. *et al.* Anti-inflammatory and analgesic activities of the latex containing triterpenes from Himatanthus sucuuba. Planta Med 66, 284–286, doi: 10.1055/s-2000-8572 (2000). 10821061

[b16] Wood C. A. *et al.* A bioactive spirolactone iridoid and triterpenoids from Himatanthus sucuuba. Chem Pharm Bull (Tokyo) 49, 1477–1478 (2001). 11724243 10.1248/cpb.49.1477

[b17] Sharma U., Singh D., Kumar P., Dobhal M. P. & Singh S. Antiparasitic activity of plumericin & isoplumericin isolated from Plumeria bicolor against Leishmania donovani. Indian J Med Res 134, 709–716, doi: 10.4103/0971-5916.91005 (2011). 22199112 PMC3249971

[b18] Kuigoua G. M. *et al.* Minor secondary metabolic products from the stem bark of Plumeria rubra Linn. displaying antimicrobial activities. Planta Med 76, 620–625, doi: 10.1055/s-0029-1240611 (2010). 19937550

[b19] Singh D. *et al.* Antifungal activity of plumericin and isoplumericin. Nat Prod Commun 6, 1567–1568 (2011). 22224260

[b20] Sollott S. J. *et al.* Taxol inhibits neointimal smooth muscle cell accumulation after angioplasty in the rat. J Clin Invest 95, 1869–1876, doi: 10.1172/JCI117867 (1995). 7706494 PMC295730

[b21] Kato J. Induction of S phase by G1 regulatory factors. Front Biosci 4, D787–792 (1999). 10577389 10.2741/kato

[b22] Sherr C. J. Cancer cell cycles. Science 274, 1672–1677 (1996). 8939849 10.1126/science.274.5293.1672

[b23] Knudsen E. S. & Wang J. Y. Dual mechanisms for the inhibition of E2F binding to RB by cyclin-dependent kinase-mediated RB phosphorylation. Mol Cell Biol 17, 5771–5783 (1997). 9315635 10.1128/mcb.17.10.5771 PMC232425

[b24] Klein E. A. & Assoian R. K. Transcriptional regulation of the cyclin D1 gene at a glance. J Cell Sci 121, 3853–3857, doi: 10.1242/jcs.039131 (2008). 19020303 PMC4545630

[b25] Mehrhof F. B., Schmidt-Ullrich R., Dietz R. & Scheidereit C. Regulation of vascular smooth muscle cell proliferation: role of NF-kappaB revisited. Circ Res 96, 958–964, doi: 10.1161/01.RES.0000166924.31219.49 (2005). 15831813

[b26] Schust J., Sperl B., Hollis A., Mayer T. U. & Berg T. Stattic: a small-molecule inhibitor of STAT3 activation and dimerization. Chem Biol 13, 1235–1242, doi: 10.1016/j.chembiol.2006.09.018 (2006). 17114005

[b27] Zgheib C. *et al.* Acyloxy nitroso compounds inhibit LIF signaling in endothelial cells and cardiac myocytes: evidence that STAT3 signaling is redox-sensitive. PLoS One 7, e43313, doi: 10.1371/journal.pone.0043313 (2012). 22905257 PMC3419695

[b28] Sobotta M. C. *et al.* Peroxiredoxin-2 and STAT3 form a redox relay for H2O2 signaling. Nat Chem Biol 11, 64–70, doi: 10.1038/nchembio.1695 (2015). 25402766

[b29] Li L. & Shaw P. E. A STAT3 dimer formed by inter-chain disulphide bridging during oxidative stress. Biochem Biophys Res Commun 322, 1005–1011, doi: 10.1016/j.bbrc.2004.08.014 (2004). 15336564

[b30] Butturini E. *et al.* S-Glutathionylation at Cys328 and Cys542 impairs STAT3 phosphorylation. ACS Chem Biol 9, 1885–1893, doi: 10.1021/cb500407d (2014). 24941337

[b31] Xie Y., Kole S., Precht P., Pazin M. J. & Bernier M. S-glutathionylation impairs signal transducer and activator of transcription 3 activation and signaling. Endocrinology 150, 1122–1131, doi: 10.1210/en.2008-1241 (2009). 18988672 PMC2654735

[b32] Butturini E. *et al.* Two naturally occurring terpenes, dehydrocostuslactone and costunolide, decrease intracellular GSH content and inhibit STAT3 activation. PLoS One 6, e20174, doi: 10.1371/journal.pone.0020174 (2011). 21625597 PMC3097233

[b33] Ghezzi P. & Di Simplicio P. Glutathionylation pathways in drug response. Curr Opin Pharmacol 7, 398–403, doi: 10.1016/j.coph.2007.04.006 (2007). 17611156

[b34] Gallogly M. M. & Mieyal J. J. Mechanisms of reversible protein glutathionylation in redox signaling and oxidative stress. Curr Opin Pharmacol 7, 381–391, doi: 10.1016/j.coph.2007.06.003 (2007). 17662654

[b35] Winterbourn C. C. & Hampton M. B. Thiol chemistry and specificity in redox signaling. Free Radic Biol Med 45, 549–561, doi: 10.1016/j.freeradbiomed.2008.05.004 (2008). 18544350

[b36] Yu C. L. *et al.* Enhanced DNA-binding activity of a Stat3-related protein in cells transformed by the Src oncoprotein. Science 269, 81–83 (1995). 7541555 10.1126/science.7541555

[b37] Liao X. H. *et al.* STAT3 Protein Regulates Vascular Smooth Muscle Cell Phenotypic Switch by Interaction with Myocardin. J Biol Chem 290, 19641–19652, doi: 10.1074/jbc.M114.630111 (2015). 26100622 PMC4528129

[b38] Sun J. *et al.* Preventing intimal thickening of vein grafts in vein artery bypass using STAT-3 siRNA. J Transl Med 10, 2, doi: 10.1186/1479-5876-10-2 (2012). 22216901 PMC3286375

[b39] Schwaiberger A. V. *et al.* Indirubin-3′-monoxime blocks vascular smooth muscle cell proliferation by inhibition of signal transducer and activator of transcription 3 signaling and reduces neointima formation *in vivo*. Arterioscler Thromb Vasc Biol 30, 2475–2481, doi: 10.1161/ATVBAHA.110.212654 (2010). 20847306

[b40] Dutzmann J., Daniel J. M., Bauersachs J., Hilfiker-Kleiner D. & Sedding D. G. Emerging translational approaches to target STAT3 signalling and its impact on vascular disease. Cardiovasc Res 106, 365–374, doi: 10.1093/cvr/cvv103 (2015). 25784694 PMC4431663

[b41] Butturini E. *et al.* Mild oxidative stress induces S-glutathionylation of STAT3 and enhances chemosensitivity of tumoural cells to chemotherapeutic drugs. Free Radic Biol Med 65, 1322–1330, doi: 10.1016/j.freeradbiomed.2013.09.015 (2013). 24095958

[b42] Baird L. & Dinkova-Kostova A. T. The cytoprotective role of the Keap1-Nrf2 pathway. Arch Toxicol 85, 241–272, doi: 10.1007/s00204-011-0674-5 (2011). 21365312

[b43] Moinova H. R. & Mulcahy R. T. Up-regulation of the human gamma-glutamylcysteine synthetase regulatory subunit gene involves binding of Nrf-2 to an electrophile responsive element. Biochem Biophys Res Commun 261, 661–668, doi: 10.1006/bbrc.1999.1109 (1999). 10441483

[b44] Liu R. *et al.* Identification and characterization of [6]-shogaol from ginger as inhibitor of vascular smooth muscle cell proliferation. Mol Nutr Food Res 59, 843–852, doi: 10.1002/mnfr.201400791 (2015). 25631547 PMC4573514

[b45] Heiss E. H. *et al.* Identification of chromomoric acid C-I as an Nrf2 activator in Chromolaena odorata. J Nat Prod 77, 503–508, doi: 10.1021/np400778m (2014). 24476568 PMC3971763

[b46] Kim S. E. *et al.* Role of Pin1 in neointima formation: down-regulation of Nrf2-dependent heme oxygenase-1 expression by Pin1. Free Radic Biol Med 48, 1644–1653, doi: 10.1016/j.freeradbiomed.2010.03.013 (2010). 20307651

[b47] Heiss E. H., Schachner D., Werner E. R. & Dirsch V. M. Active NF-E2-related factor (Nrf2) contributes to keep endothelial NO synthase (eNOS) in the coupled state: role of reactive oxygen species (ROS), eNOS, and heme oxygenase (HO-1) levels. J Biol Chem 284, 31579–31586, doi: 10.1074/jbc.M109.009175 (2009). 19797052 PMC2797228

[b48] Chen X. L. *et al.* Activation of Nrf2/ARE pathway protects endothelial cells from oxidant injury and inhibits inflammatory gene expression. Am J Physiol Heart Circ Physiol 290, H1862–1870, doi: 10.1152/ajpheart.00651.2005 (2006). 16339837

[b49] Rodriguez A. I. *et al.* HO-1 and CO decrease platelet-derived growth factor-induced vascular smooth muscle cell migration via inhibition of Nox1. Arterioscler Thromb Vasc Biol 30, 98–104, doi: 10.1161/ATVBAHA.109.197822 (2010). 19875720 PMC2814251

[b50] Liu Z. *et al.* Tanshinone IIA suppresses cholesterol accumulation in human macrophages: role of heme oxygenase-1. J Lipid Res 55, 201–213, doi: 10.1194/jlr.M040394 (2014). 24302760 PMC3886659

[b51] Banning A. & Brigelius-Flohe R. NF-kappaB, Nrf2, and HO-1 interplay in redox-regulated VCAM-1 expression. Antioxid Redox Signal 7, 889–899, doi: 10.1089/ars.2005.7.889 (2005). 15998244

[b52] Fitzpatrick L. R., Stonesifer E., Small J. S. & Liby K. T. The synthetic triterpenoid (CDDO-Im) inhibits STAT3, as well as IL-17, and improves DSS-induced colitis in mice. Inflammopharmacology 22, 341–349, doi: 10.1007/s10787-014-0203-2 (2014). 24715223

[b53] Liby K. *et al.* The synthetic triterpenoids, CDDO and CDDO-imidazolide, are potent inducers of heme oxygenase-1 and Nrf2/ARE signaling. Cancer Res 65, 4789–4798, doi: 10.1158/0008-5472.CAN-04-4539 (2005). 15930299

[b54] Yore M. M., Liby K. T., Honda T., Gribble G. W. & Sporn M. B. The synthetic triterpenoid 1-[2-cyano-3,12-dioxooleana-1,9(11)-dien-28-oyl]imidazole blocks nuclear factor-kappaB activation through direct inhibition of IkappaB kinase beta. Mol Cancer Ther 5, 3232–3239, doi: 10.1158/1535-7163.MCT-06-0444 (2006). 17148759

[b55] Seidel P. *et al.* Dimethylfumarate inhibits NF-{kappa}B function at multiple levels to limit airway smooth muscle cell cytokine secretion. Am J Physiol Lung Cell Mol Physiol 297, L326–339, doi: 10.1152/ajplung.90624.2008 (2009). 19465513

[b56] Ashrafian H. *et al.* Fumarate is cardioprotective via activation of the Nrf2 antioxidant pathway. Cell Metab 15, 361–371, doi: 10.1016/j.cmet.2012.01.017 (2012). 22405071 PMC3314920

[b57] Kang H. J. *et al.* Dimethylfumarate suppresses adipogenic differentiation in 3T3-L1 preadipocytes through inhibition of STAT3 activity. PLoS One 8, e61411, doi: 10.1371/journal.pone.0061411 (2013). 23637829 PMC3630208

[b58] Bharti A. C., Donato N. & Aggarwal B. B. Curcumin (diferuloylmethane) inhibits constitutive and IL-6-inducible STAT3 phosphorylation in human multiple myeloma cells. J Immunol 171, 3863–3871 (2003). 14500688 10.4049/jimmunol.171.7.3863

[b59] Singh S. & Aggarwal B. B. Activation of transcription factor NF-kappa B is suppressed by curcumin (diferuloylmethane) [corrected]. J Biol Chem 270, 24995–25000 (1995). 7559628 10.1074/jbc.270.42.24995

[b60] Balogun E. *et al.* Curcumin activates the haem oxygenase-1 gene via regulation of Nrf2 and the antioxidant-responsive element. Biochem J 371, 887–895, doi: 10.1042/BJ20021619 (2003). 12570874 PMC1223348

[b61] Pastore A. & Piemonte F. S-Glutathionylation signaling in cell biology: progress and prospects. Eur J Pharm Sci 46, 279–292, doi: 10.1016/j.ejps.2012.03.010 (2012). 22484331

[b62] Saengsai J., Kongtunjanphuk S., Yoswatthana N., Kummalue T. & Jiratchariyakul W. Antibacterial and Antiproliferative Activities of Plumericin, an Iridoid Isolated from Momordica charantia Vine. Evid Based Complement Alternat Med 2015, 823178, doi: 10.1155/2015/823178 (2015). 25945113 PMC4405293

[b63] Bauer R. A. Covalent inhibitors in drug discovery: from accidental discoveries to avoided liabilities and designed therapies. Drug Discov Today, doi: 10.1016/j.drudis.2015.05.005 (2015). 26002380

[b64] Zimmermann K. *et al.* Activated AMPK boosts the Nrf2/HO-1 signaling axis-A role for the unfolded protein response. Free Radic Biol Med, doi: 10.1016/j.freeradbiomed.2015.03.030 (2015). PMC4568300 25843659

[b65] Griffith O. W. Determination of glutathione and glutathione disulfide using glutathione reductase and 2-vinylpyridine. Anal Biochem 106, 207–212 (1980). 7416462 10.1016/0003-2697(80)90139-6

[b66] Gerhauser C. *et al.* Cancer chemopreventive potential of sulforamate, a novel analogue of sulforaphane that induces phase 2 drug-metabolizing enzymes. Cancer Res 57, 272–278 (1997). 9000567

[b67] Rahman I., Kode A. & Biswas S. K. Assay for quantitative determination of glutathione and glutathione disulfide levels using enzymatic recycling method. Nat Protoc 1, 3159–3165, doi: 10.1038/nprot.2006.378 (2006). 17406579

