# Supplementary material for: Plumericin inhibits proliferation of vascular smooth muscle cells by blocking STAT3 signaling via S-glutathionylation
Source: Sci Rep. 2016 Feb 9;6:20771. doi: 10.1038/srep20771 (PMC4746734; doi:10.1038/srep20771)
Supplement: Supplementary Information [file srep20771-s1.pdf]

Supplemental Information

Plumericin inhibits proliferation of vascular smooth muscle cells by blocking STAT3 signaling via *S*-glutathionylation

Elke H Heiss[1,*,#], Rongxia Liu[1,2,#], Birgit Waltenberger[3], Shafaat Khan[4,5], Daniel Schachner[1], Paul Kollmann[1], Kristin Zimmermann[1], Muris Cabaravdic[4], Pavel Uhrin[4], Hermann Stuppner[3], Johannes M Breuss[4], Atanas G Atanasov[1*] and Verena M Dirsch[1]

[1] *Department of Pharmacognosy, University of Vienna, Althanstrasse 14, 1090 Vienna, Austria*

[2] *Current address: School of Pharmacy, Yantai University, Yantai, China*

[3] *Institute of Pharmacy (Pharmacognosy) and Center for Molecular Biosciences Innsbruck (CMBI), University of Innsbruck, Innsbruck, Austria*

[4] *Center for Physiology and Pharmacology, Institute for Vascular Biology and Thrombosis Research, Medical University of Vienna, Vienna, Austria*

[5] *Department of Zoology, University of Sargodha, Sargodha, Pakistan*

Material and Methods

Reagents, antibodies, and cells

Wortmannin, U0126 and SP600125 SB203580 were purchased from Tocris (Bristol, UK). Antibodies against phospho-Akt (Ser473), phospho-Erk1/2 MAPK (Thr202/Tyr204), phospho-p38MAPK (Thr180/Tyr182), phospho-JNK (Thr183/Tyr185), Akt, Erk1/2, p38MAPK, JNK, and IκB-α were purchased from New England Biolabs (Frankfurt, Germany). The HO-1 inhibitor tin protoporphyrin IX dichloride (SnPP) as well as the HO-1 antibody were from Enzo Life Sciences (Lausen, Switzerland). The immortalized human umbilical vein endothelial cell line (HUVECtert) was kindly provided by Dr. Hannes Stockinger (Medical University of Vienna, Austria).[1] HUVECtert were grown in endothelial cell basal medium (EBM) supplemented with 10 % FCS, 100 U/mL penicillin, 100 µg/mL streptomycin, 1 % amphotericin B and EBM SingleQuots®, containing recombinant human epidermal growth factor, hydrocortisone, gentamicin sulfate, amphotericin B, and 0.4 % bovine brain extract. The stably transfected CHO-ARE LUC Nrf2-responsive reporter cell line was established and cultivated as reported previously. [2]

Resazurin conversion assay

VSMC were seeded at a density of 5 × 10^3 cells/well in 96-well plates. 24 h later, cells were serum-deprived for 24 h to render them quiescent. Plumericin or vehicle (0.1 % DMSO) was added as indicated, and after 30 min of preincubation, the cells were stimulated with NBS (10 % final concentration) for 48 h. To measure the number of metabolically active VSMC by conversion of resazurin to fluorescent resorufin[3], cells were washed with phosphate-buffered saline (PBS) and incubated in serum-free medium containing 10 µg/mL resazurin for 2 h. Samples were measured by monitoring the increase in fluorescence at a wavelength of 590 nm using an excitation wavelength of 535 nm in a 96-well plate reader (Tecan GENios Pro). HUVECtert cells [1] were cultivated at 5 × 10^3 cells/well in 96-well plates. After 24 h, HUVECtert cells were treated with either plumericin, the positive control taxol (1 µM), or vehicle (0.1% DMSO) as indicated, and incubated for 48 h. Then, cells

were washed with PBS and incubated in culture medium containing 10 µg/mL resazurin for 2 h. The detection step was performed as described above.

Crystal violet biomass staining

VSMC were seeded at a density of 5 × 10^3 cells/well in 96-well plates. After 24 h, cells were serum-deprived for 24 h to render them quiescent. Afterwards, plumericin or vehicle (0.1 % DMSO) was added as indicated, and after preincubation for 30 min, cells were stimulated with NBS (10 % final concentration) for 48 h. To determine the total biomass, cells were incubated in 100 µL crystal violet staining solution (0.5 % crystal violet, 20 % methanol) for 15 min and then thoroughly washed with ddH_2O. After drying, 100 µL EtOH/sodium citrate solution were added (EtOH: 0.1M sodium citrate = 1:1) and the absorbance of the samples was measured at 595 nm in a 96-well plate reader (Tecan sunrise).

Antioxidant response element (ARE)-dependent luciferase (LUC) reporter gene assay

CHO-ARE-LUC cells were seeded into 96-well plates and treated with solvent (0.1 % DMSO) or the indicated concentrations of plumericin for 16 h before luciferase activity was assessed and normalized to fluorescence from co-transfected enhanced green fluorescent protein (EGFP) as described elsewhere.[4]

Murine femoral artery cuff model

Male C57BL/6J mice at 8 to 12 weeks of age were subjected to surgical implantation of cuffs around the femoral arteries. The surgical procedure was performed as follows. Mice were anesthetized by intra-peritoneal injection of ketamine (100 mg/kg) and xylazine (10 mg/kg) and the left femoral artery of mice was exposed for 3 mm length from its surrounding tissue. Drug releasing cuffs, made from poly-(ε-caprolactone) and polyethylene glycol (PEG) (with or without addition of plumericin) were placed to surround the artery. In case of the treatment, plumericin was pre-mixed with PEG and then this mixture was combined with the molten poly-(ε-caprolactone) (exposed to 70°C) in

proportion of 1:4, for the final concentration of plumericin (165 µM) in the cuff material. We then shaped 2-3 mm long cuffs from this material to have a 0.5 mm inner diameter, and a 1 mm outer diameter. In control cuffs we omitted the plumericin supplementation. During the following 2 – 3 days mice received Tramadol (10 – 20 mg/kg, Grünenthal GmbH, Aachen, Germany) as analgesic drug. The arteries were harvested 28 d after placing of the cuff. To this, mice were anesthetized and subsequently perfused by cardiac puncture with EDTA-PBS (1.75 mg/mL). Upon perfusion, a 2 cm longitudinal incision was made in the internal side of the leg, and the cuffed femoral artery was removed and embedded for cryo-sectioning and following histological analysis. Animal care and all experimental procedures were approved by the Animal Experimental Committee of the Medical University of Vienna and by the Austrian Ministry of Science (license no. BMWF-66.009/0117-II/3b/2012).

Figure Legends

Supplemental Figure 1: Plumericin reduces metabolic activity and biomass of serum-stimulated VSMC and does not alter cellular morphology. Quiescent VSMC were treated as indicated for 30 min and then stimulated with serum (10 % NBS) for 48 h before they were subjected to a resazurin conversion assay (a) or crystal violet staining (b). Bars depict compiled data from three independent experiments (mean + SD,* $p<0.05$ (vs serum stimulated control); ANOVA, Bonferroni). (c) Microscopic pictures of VSMC treated as indicated.

Supplemental Figure 2: Plumericin arrests VSMC in G0/G1 of the cell cycle. Serum-deprived quiescent VSMC (a) were pretreated with solvent (b) or plumericin (1 µM (c) and 3 µM (d)) as indicated for 30 min and then exposed to serum (10 % NBS; b, c, and d) for 16 h before cell nuclei were stained with propidium iodide and analyzed by flow cytometry. Representative histogram plots are shown.

Supplemental Figure 3: Plumericin does not inhibit serum-induced ERK or Akt phosphorylation, but elevates p38 and JNK phosphorylation. (a) Quiescent VSMC were pretreated with 3 µM plumericin

(Plum), 1 µM wortmannin (W, PI3K inhibitor), or 20 µM UO126 (U, MEK inhibitor) for 30 min prior to exposure to serum (10 % NBS) for 10 min. Total cell lysates were subjected to immunoblot analysis for phospho-ERK1/2 (Thr202/Tyr204), ERK1/2, phospho-Akt (Ser 473), Akt, and actin. Experiments were performed three times, representative blots and compiled densitometric analyses are depicted. (mean +SD, *$p<0.05$ (compared groups are indicated by lines), ANOVA, Bonferroni). Quiescent VSMC were pretreated with 3 µM plumericin (Plum), 50 µM forskolin (F, p38 inhibitor), or 50 µM SP600125 (SP, JNK inhibitor) for 30 min prior to exposure to serum (10 % NBS) for 10 min. Total cell lysates were subjected to immunoblot analysis for (b) phospho-p38 (Thr180/Tyr 182), p38, and actin or (c) phospho-JNK (Thr183/Tyr185), JNK, and actin. Experiments were performed three times, representative blots and compiled densitometric analyses are depicted. (mean +SD, *$p<0.05$ (compared groups are indicated by lines), ANOVA, Bonferroni).

Supplemental Figure 4: Inhibition of p38 and JNK cannot overcome the plumericin-induced inhibition of VSMC proliferation. Quiescent VSMC were pretreated with 20 µM SB203580 (p38 inhibitor) (a) or 50 µM SP600125 (b) for 1 h before plumericin (1 µM) and 30 min later finally serum (10 % NBS) were added for 24 hours. Cell proliferation was assessed by a BrdU incorporation. Bar graphs depict compiled data of three independent experiments (mean+ SD, *$p<0.05$ (compared groups are indicated by lines); ANOVA, Bonferroni).

Supplemental Figure 5: IκB degradation does not take place in serum-stimulated VSMC, and plumericin has no effect on IκB levels. (a) Quiescent VSMC were stimulated with serum (10 % NBS) for the indicated periods of time before total cell lysates were subjected to western blot analysis for IκB and actin. (b) Quiescent VSMC were pretreated with plumericin (3 µM, Plum) for 30 min and then stimulated with serum (10 % NBS) for 10 min before total cell lysates were subjected to western blot analysis for IκB and actin. Representative blots and compiled densitometric data of three experiments are depicted (mean + SD).

Supplemental Figure 6: Exogenously added GSH (in form of its ester, GEE) reduces the plumericin mediated inhibition of cell cycle progression in serum-treated VSMC. Quiescent VSMC were treated with 2 mM GEE for 18 h as indicated and thoroughly washed. Cells were then treated with solvent (DMSO, 0.1 %, (-)) or plumericin (Plum, 3 µM, (+)) for 30 min and stimulated with 10 % serum for 16 h before cell nuclei were stained with PI and analyzed by flow cytometry. Data from two independent experiments were compiled and depicted as a bar graph.

Supplemental Figure 7: Plumericin appears capable of reducing neointima formation in a murine femoral cuff model and hardly influences the metabolic activity of endothelial cells. (a) Non-constrictive poly-(ε-caprolactone) and polyethylene glycol (PEG) cuffs, containing or not-containing plumericin, were placed loosely around the right femoral artery of mice. The bar graph depicts the resulting neointima (→) to media (--o) cross-sectional ratio at day 28 after cuff placement (mean + SD; n = 3; * $p < 0.05$ Student´s t test). Representative histological images of femoral arteries are depicted.(b) Immortalized HUVECtert were treated with the indicated concentrations of plumericin or taxol as a positive control for 48 h before a resazurin conversion assay was performed to assess cell viability (metabolic activity via resazurin conversion). (n=3, mean + SD., *<0.05 (vs vehicle-treated control cells); ANOVA, Bonferroni)

Supplemental Figure 8: Plumericin is able to activate Nrf2 in a reporter gene assay and triggers HO-1 expression in VSMC, however, with no causal relation to its antiproliferative activity. (a) CHO-ARE-Luc cells were treated with the indicated concentrations of plumericin for 16 h before luciferase activity as measure for Nrf2 activation was assessed. The bar graph depicts fold activation compared to the DMSO control (mean + SD, n=2). (b) Quiescent VSMC were pretreated with 3 µM plumericin (Plum) for 30 min and then exposed to serum (10 % NBS) as indicated. After lysis, total cell lysates were subjected to immunoblot analysis for HO-1 and actin. Representative blots of three independent experiments are depicted. (c) Quiescent VSMC were pretreated with 40 µM SnPP (a HO-1 inhibitor) for 1 h before plumericin (1 µM) and 30 min later finally serum (10 % NBS) were added for 24 h. Cell proliferation was assessed by a BrdU incorporation assay. Bar graphs depict compiled

data of three independent experiments (mean + SD, *p<0.05 (compared groups are indicated by lines); ANOVA, Bonferroni).

Supplemental Figure 9: Original Western Blot Images

References

1 Schiller, H. B., Szekeres, A., Binder, B. R., Stockinger, H. & Leksa, V. Mannose 6-phosphate/insulin-like growth factor 2 receptor limits cell invasion by controlling alphaVbeta3 integrin expression and proteolytic processing of urokinase-type plasminogen activator receptor. *Mol Biol Cell* **20**, 745-756, doi:10.1091/mbc.E08-06-0569 (2009).

2 Kropat, C. *et al.* Modulation of Nrf2-dependent gene transcription by bilberry anthocyanins in vivo. *Mol Nutr Food Res* **57**, 545-550, doi:10.1002/mnfr.201200504 (2013).

3 Joa, H. *et al.* Identification of ostruthin from Peucedanum ostruthium rhizomes as an inhibitor of vascular smooth muscle cell proliferation. *J Nat Prod* **74**, 1513-1516, doi:10.1021/np200072a (2011).

4 Heiss, E. H. *et al.* Identification of chromomoric acid C-I as an Nrf2 activator in Chromolaena odorata. *J Nat Prod* **77**, 503-508, doi:10.1021/np400778m (2014).

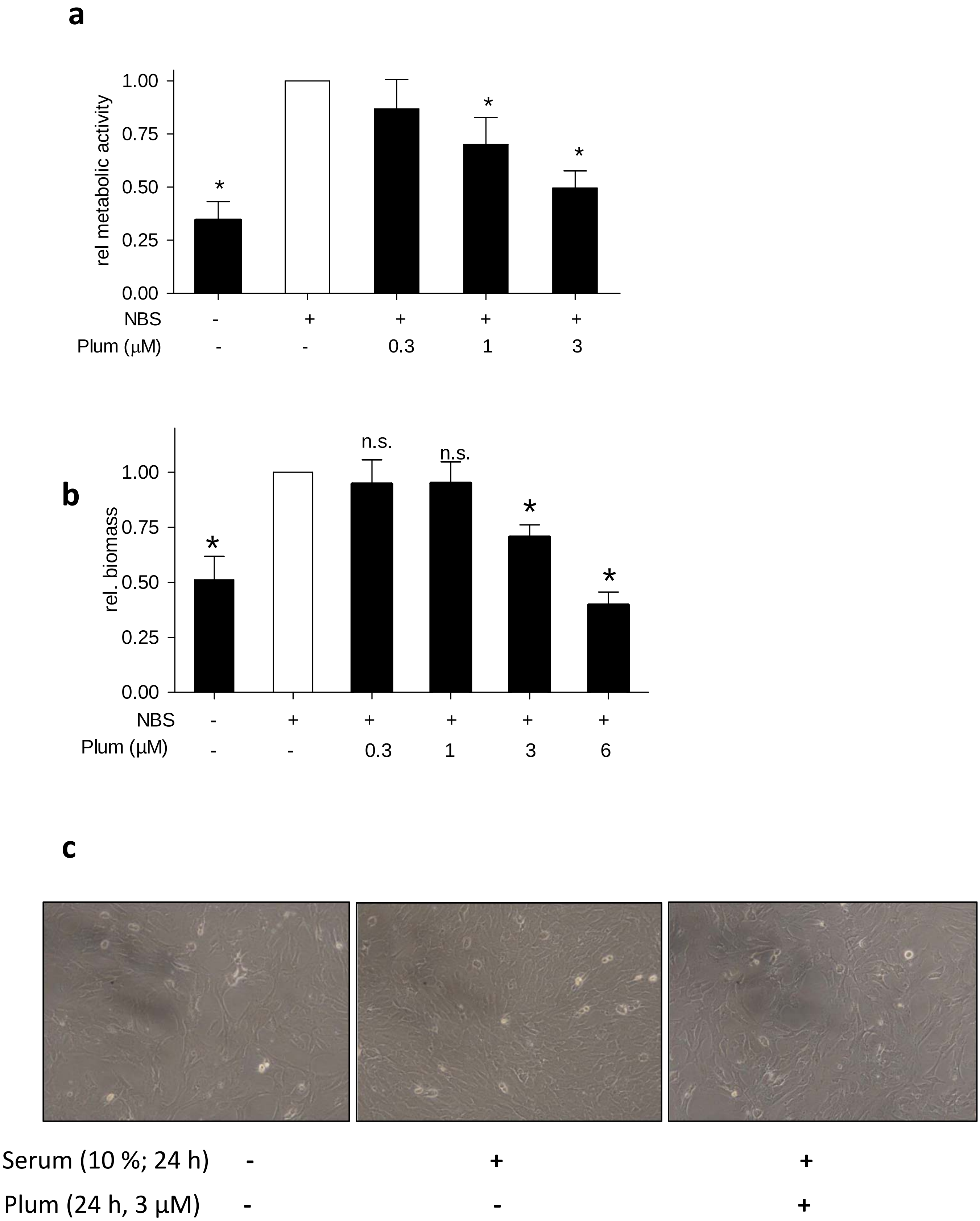

Supplemental Figure 1

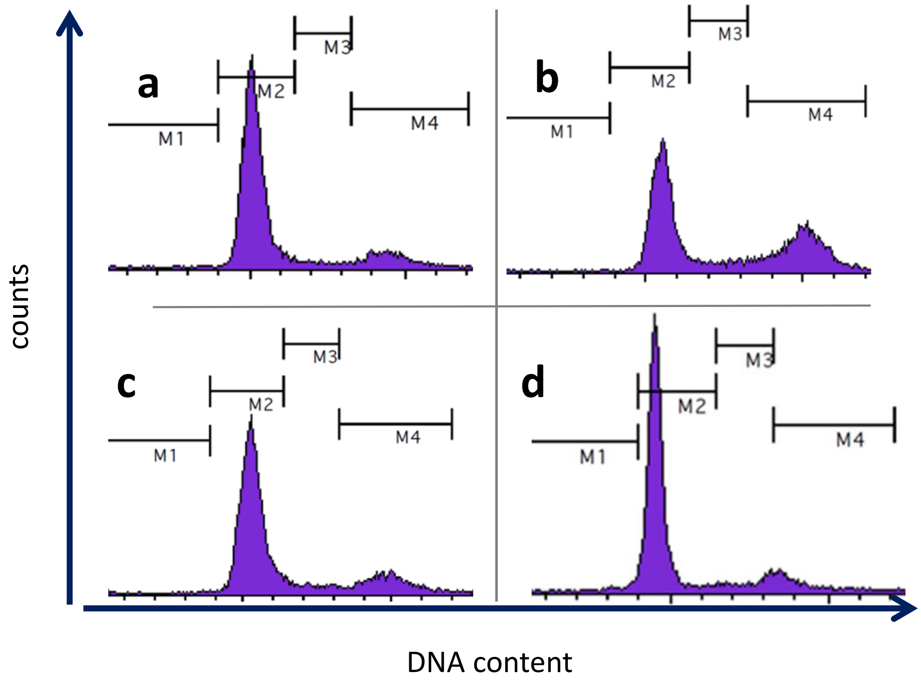

Supplemental Figure 2

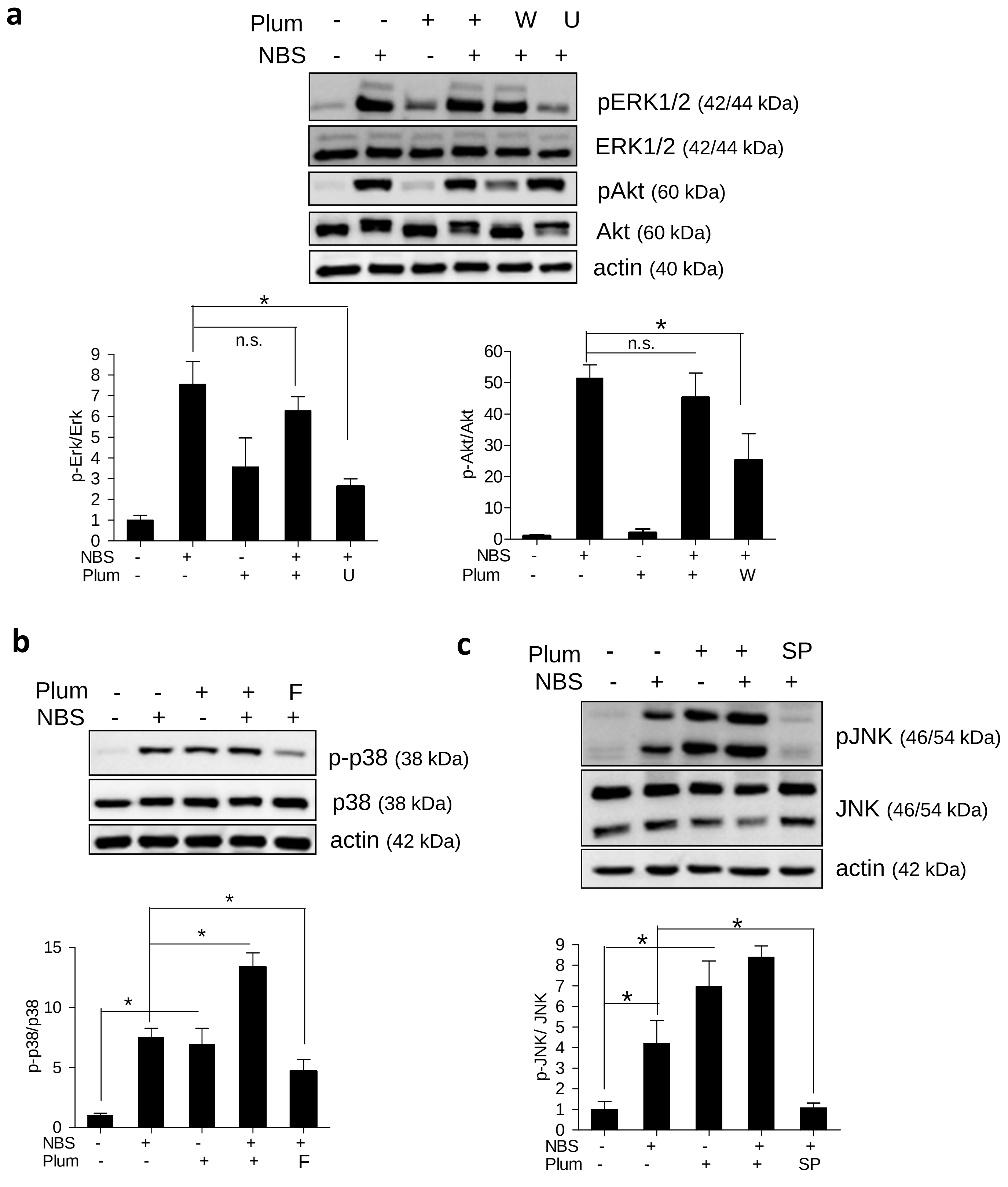

Supplemental Figure 3

a

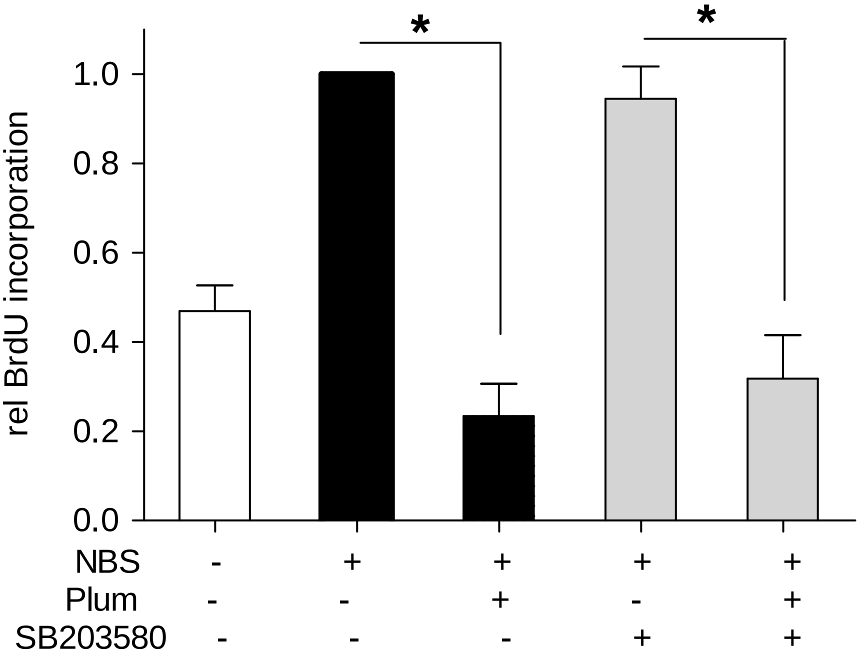

b

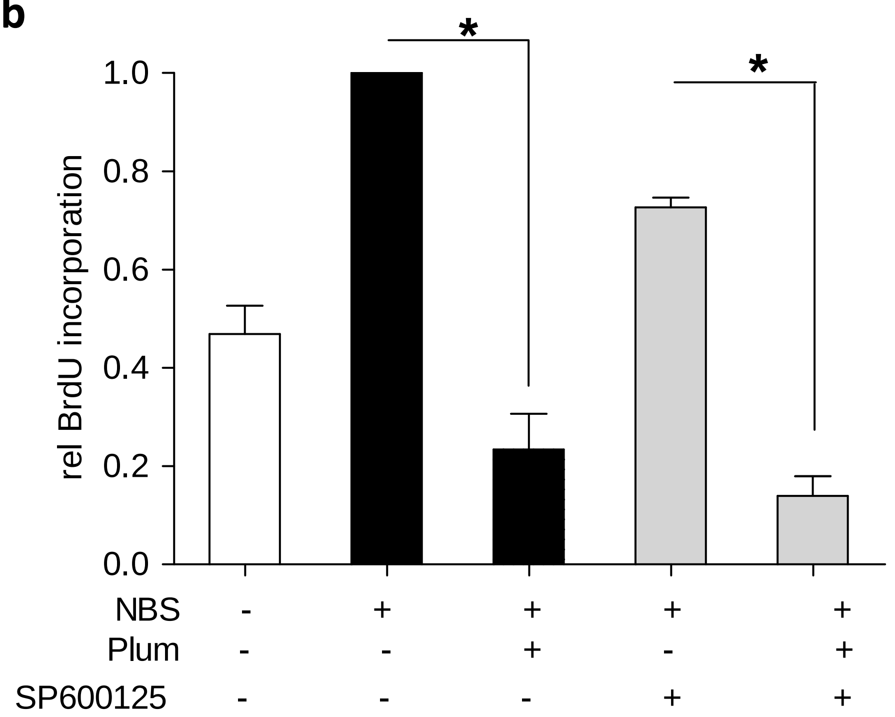

Supplemental Figure 4

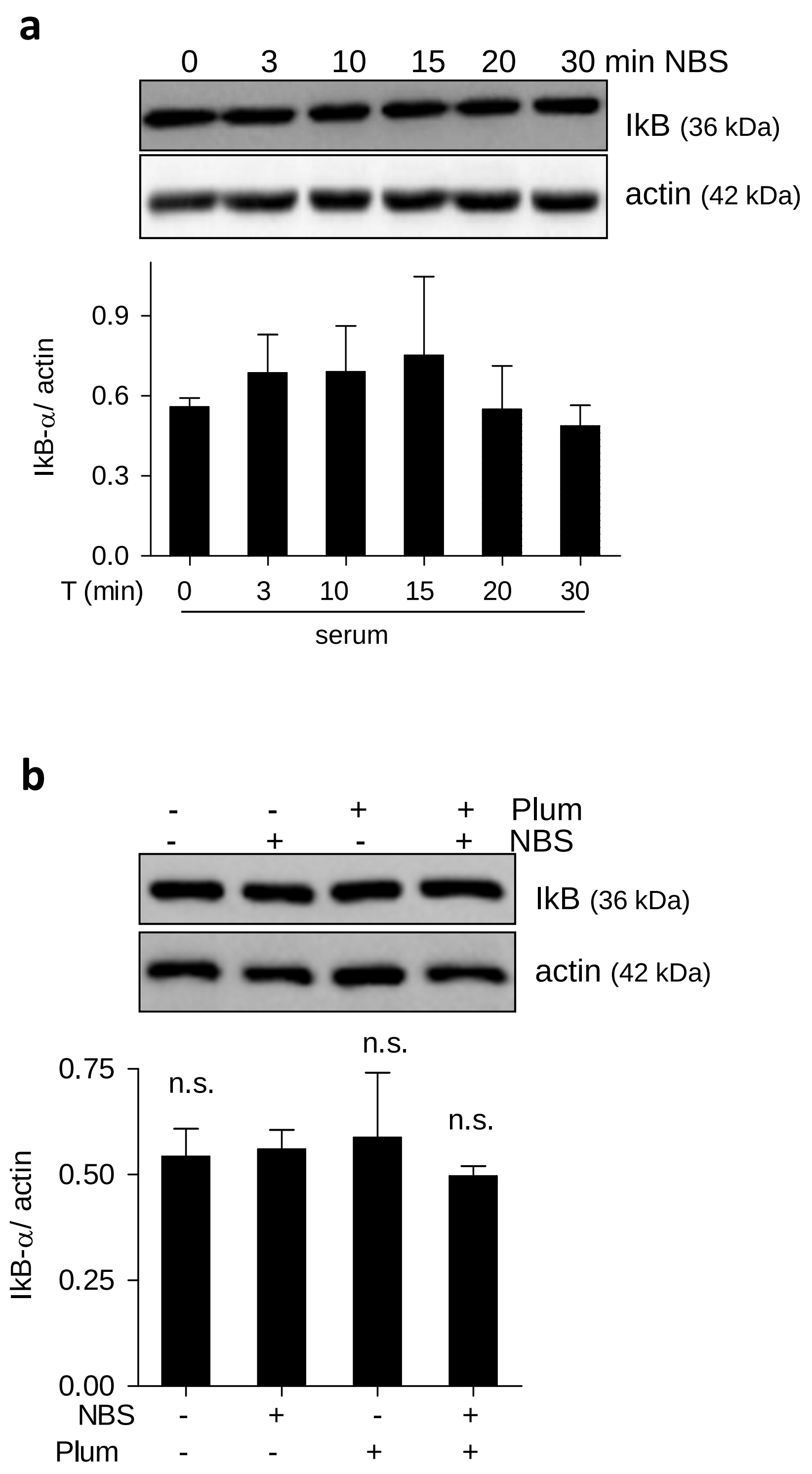

Supplemental Figure 5

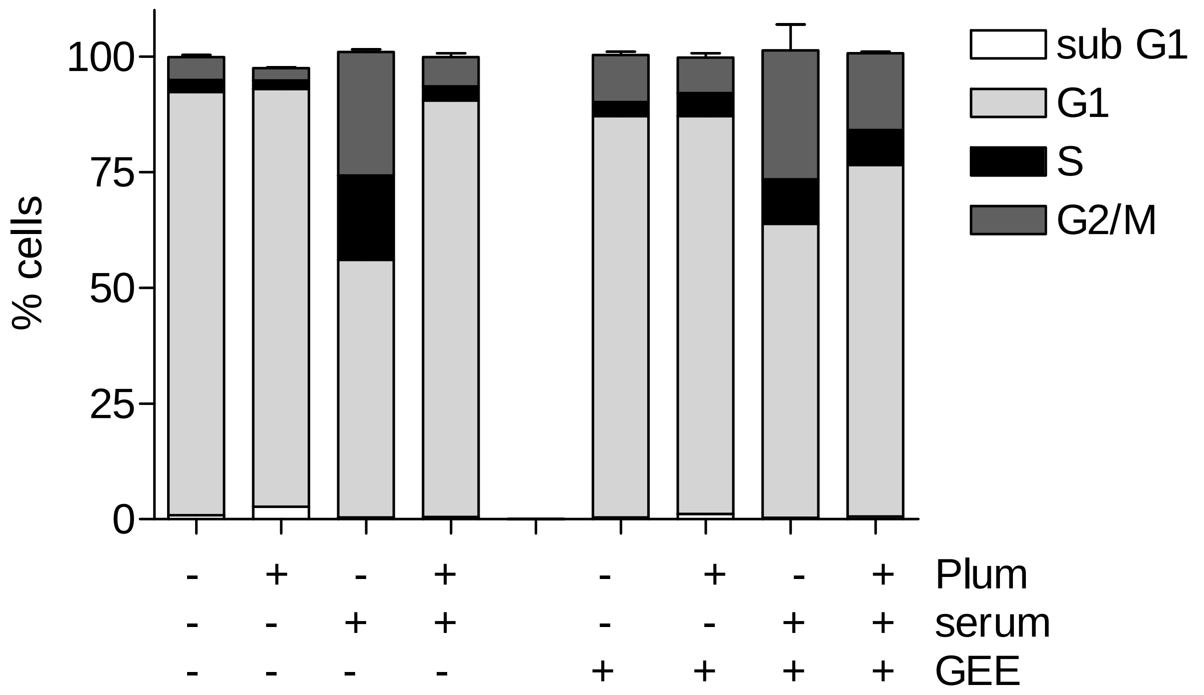

Supplemental Figure 6

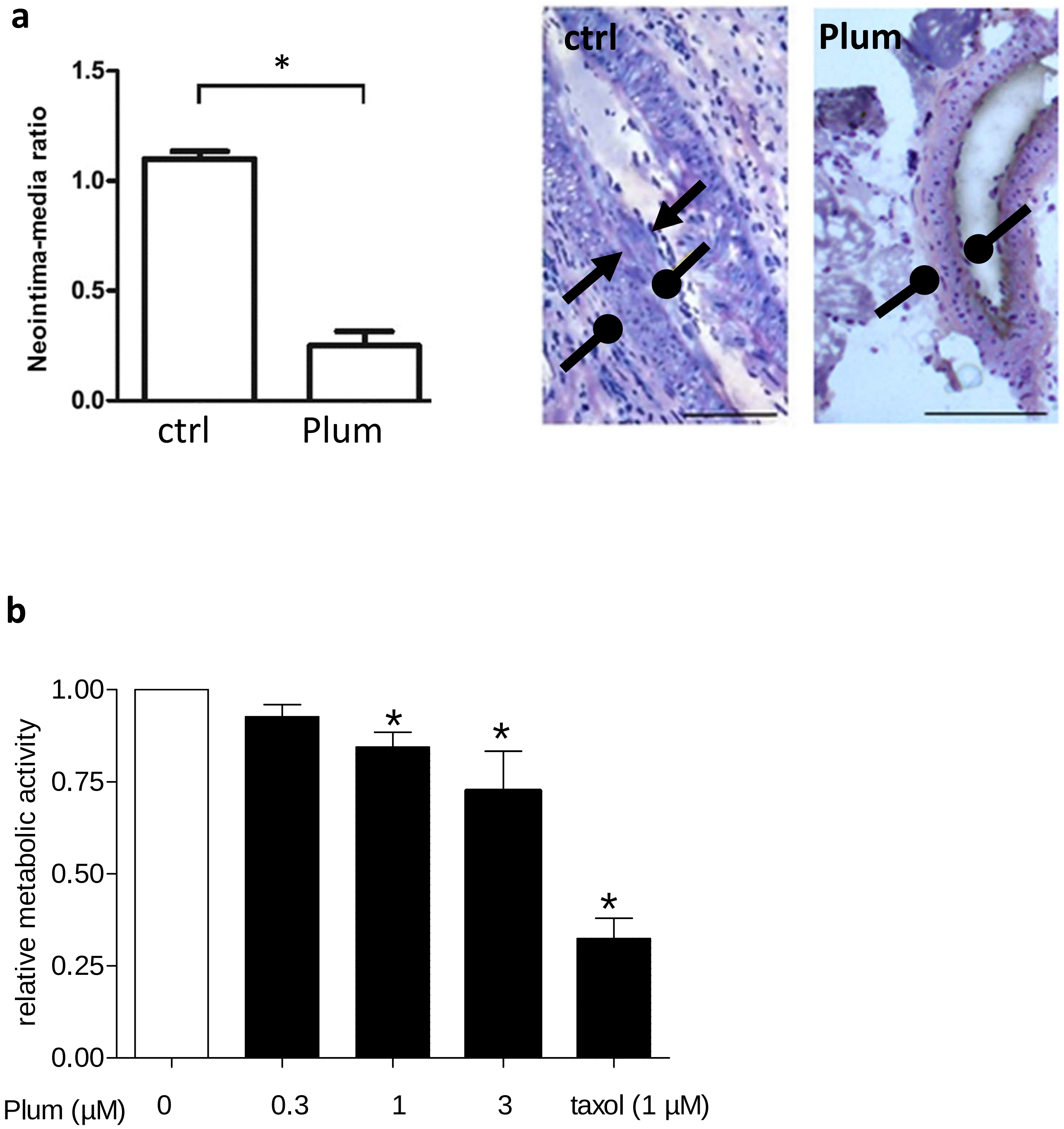

Supplemental Figure 7

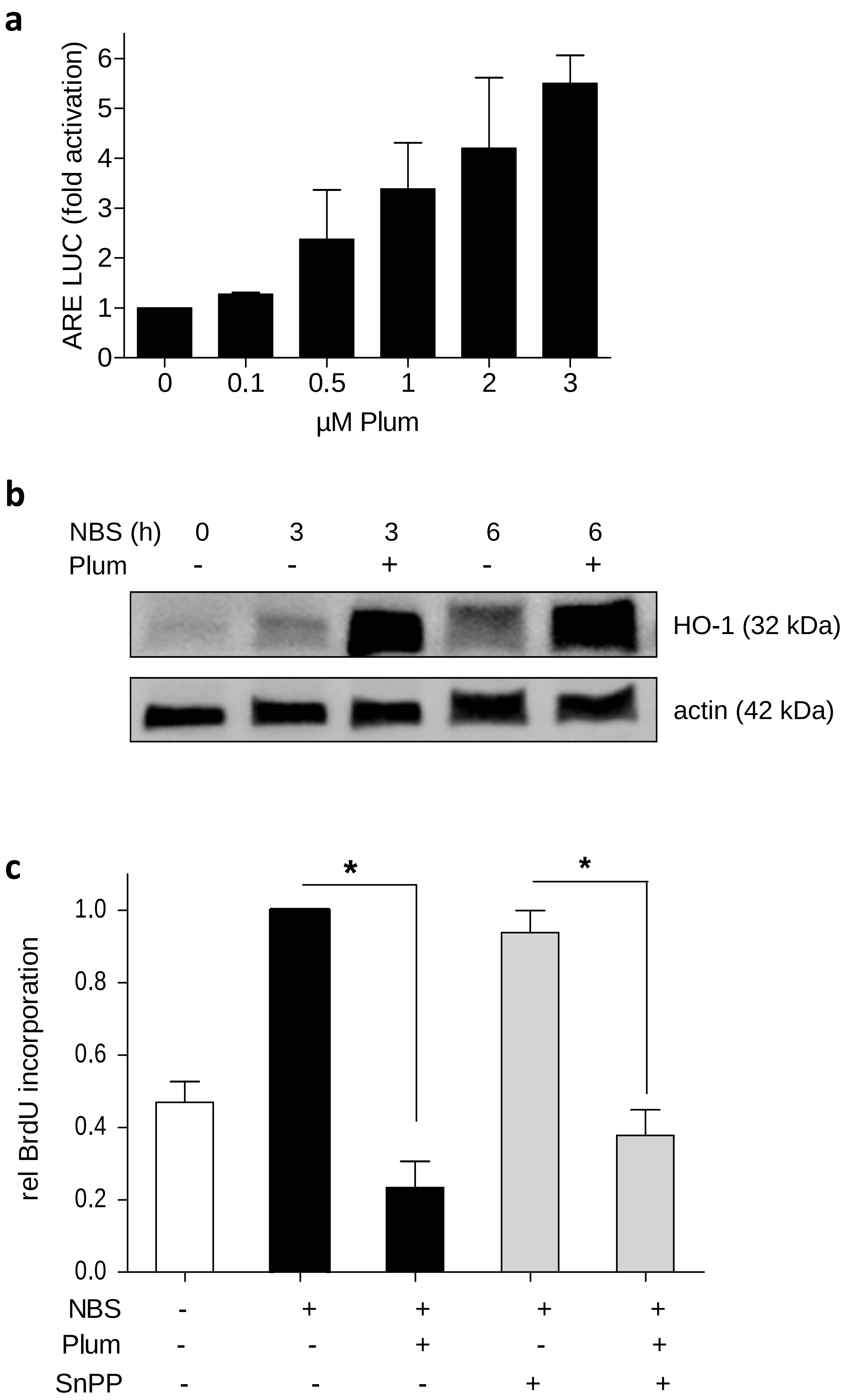

Supplemental Figure 8

Supplemental Figure 9

Original Western Blot Images

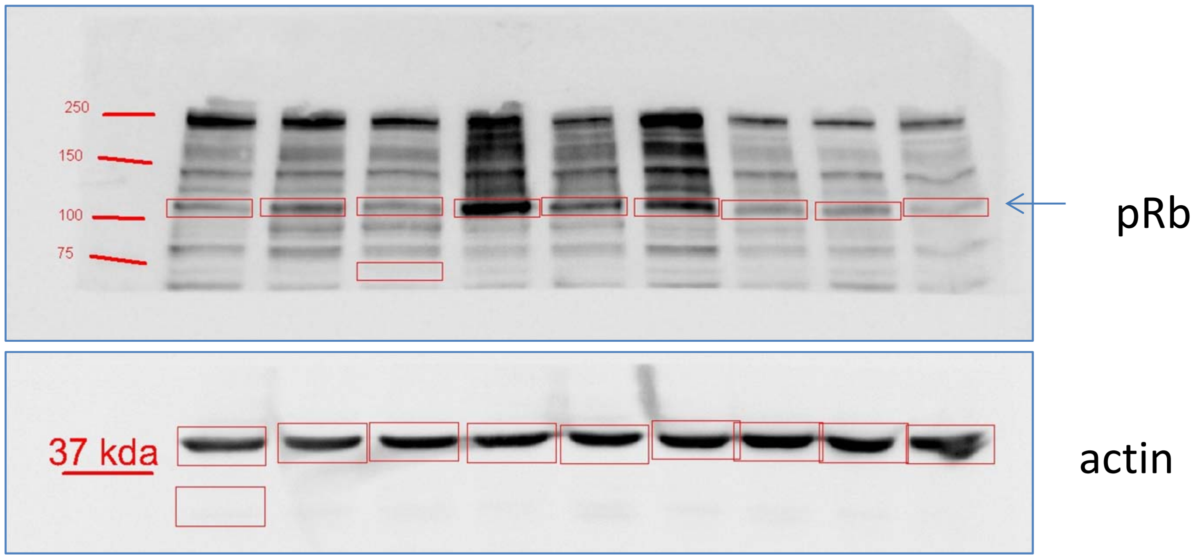

Representative Blots for Figure 2 b

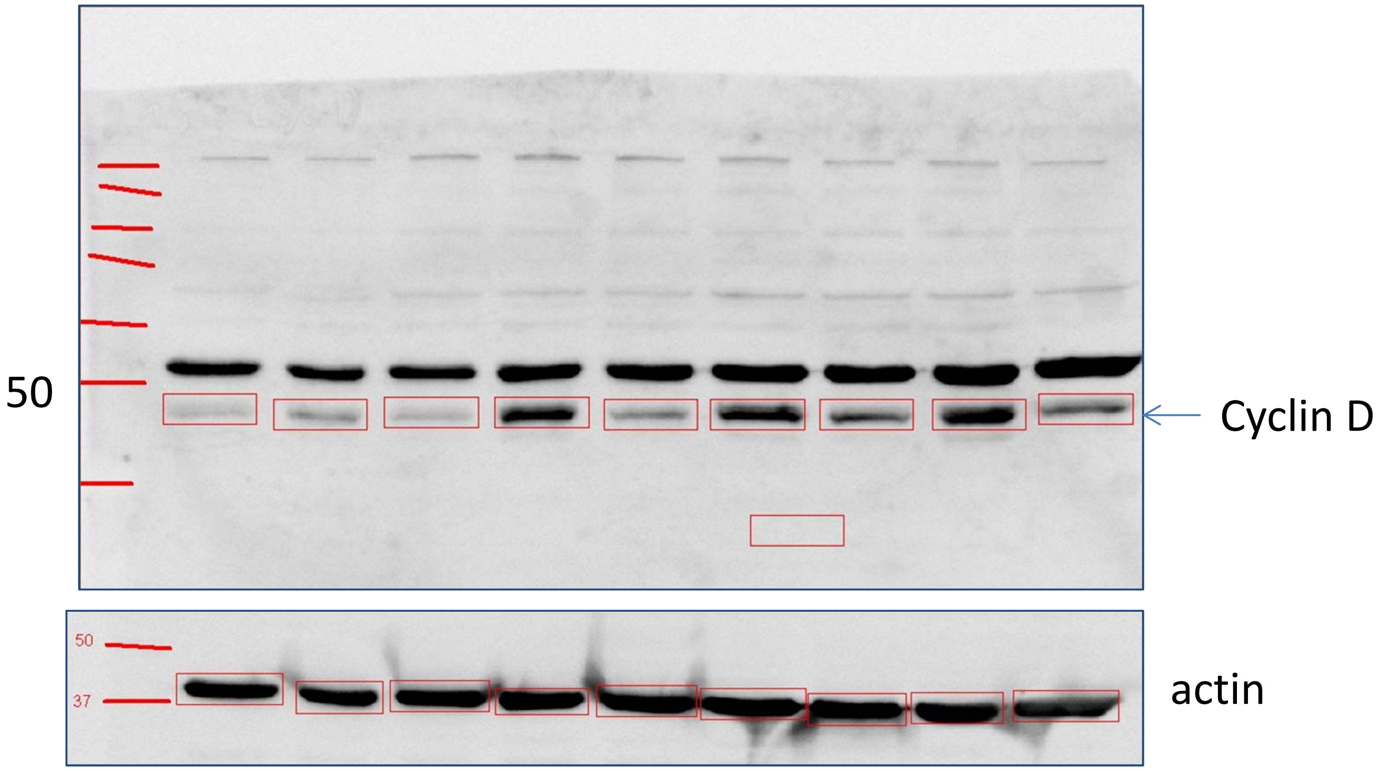

Representative Blots for Figure 3 a

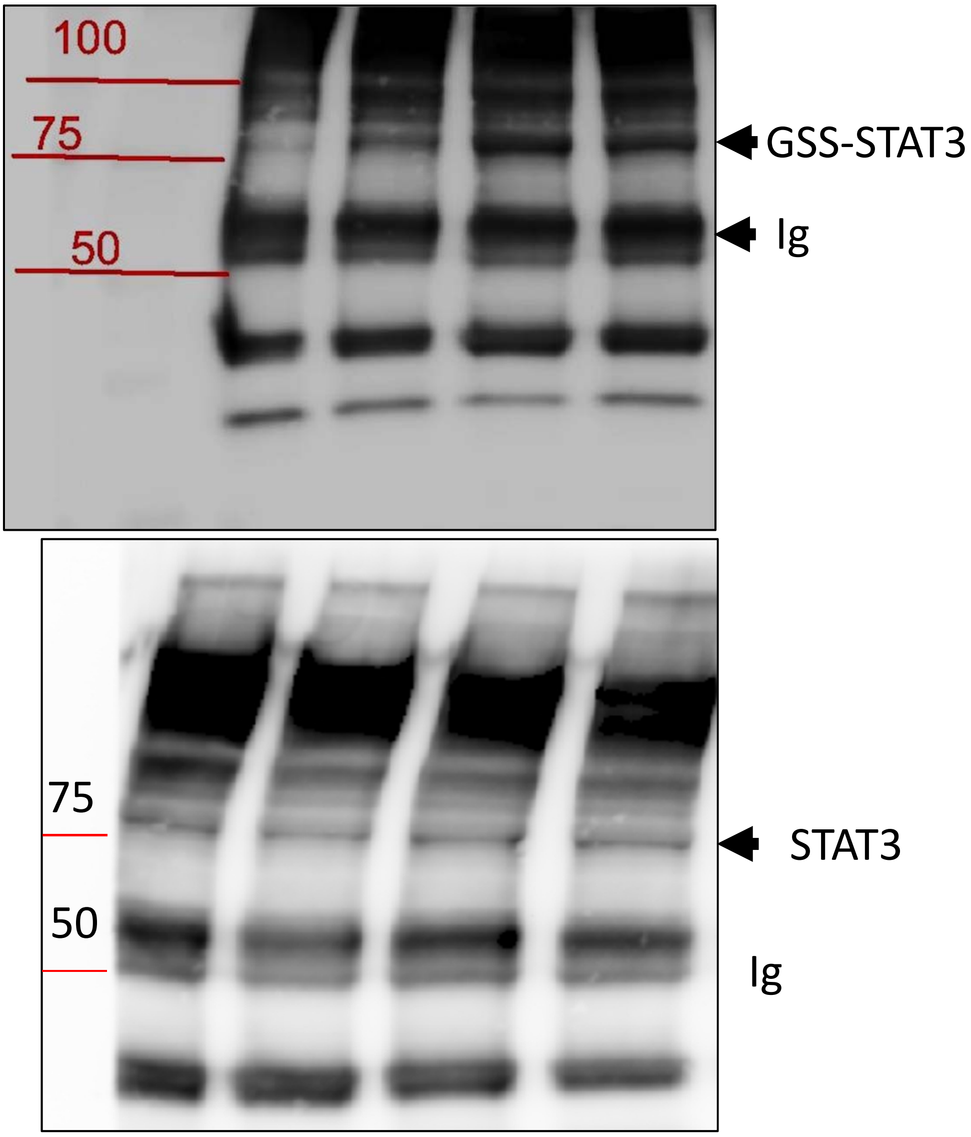

Representative Blots for Figure 5 a

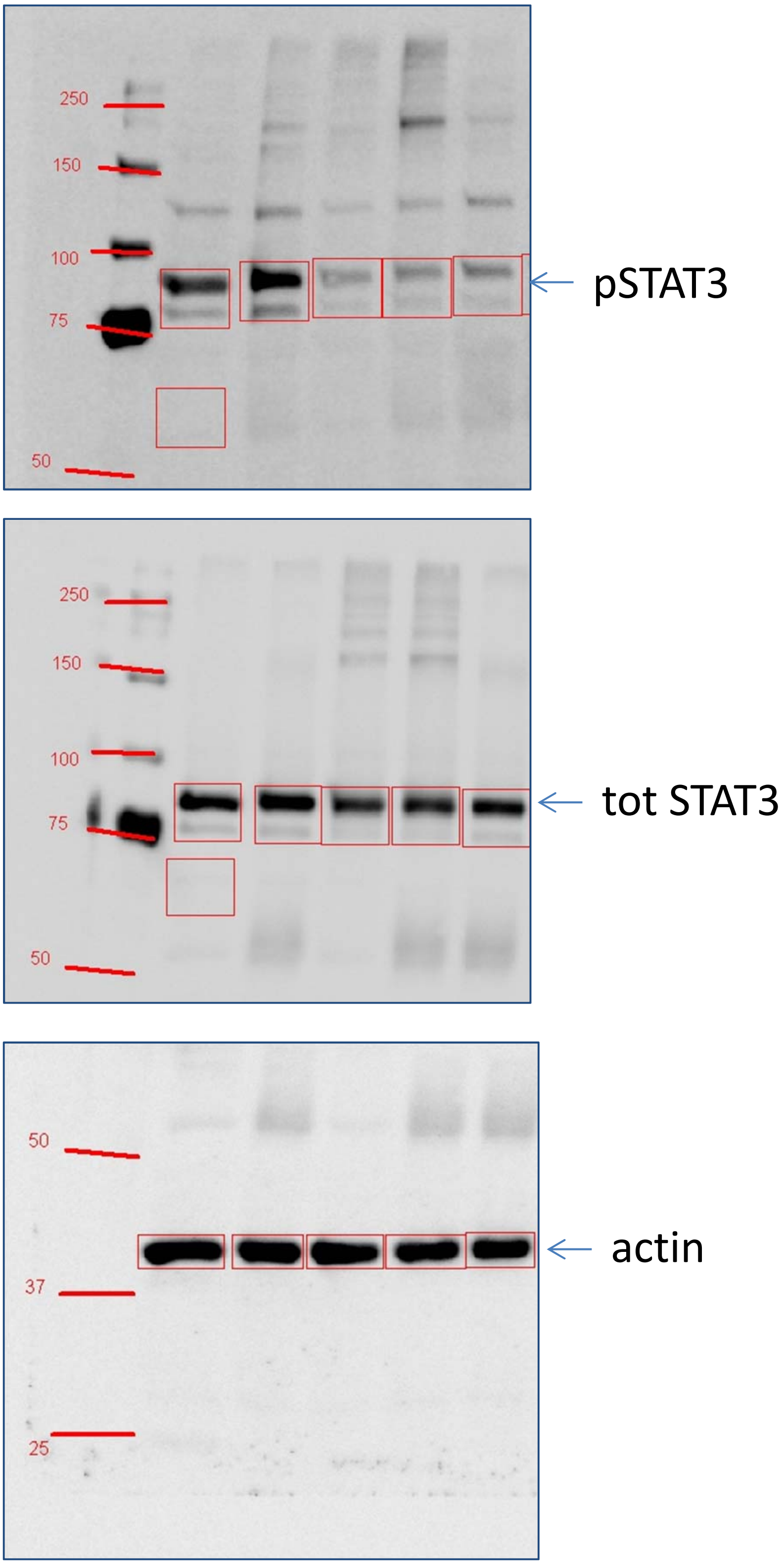

Representative Blots for Figure 5 b

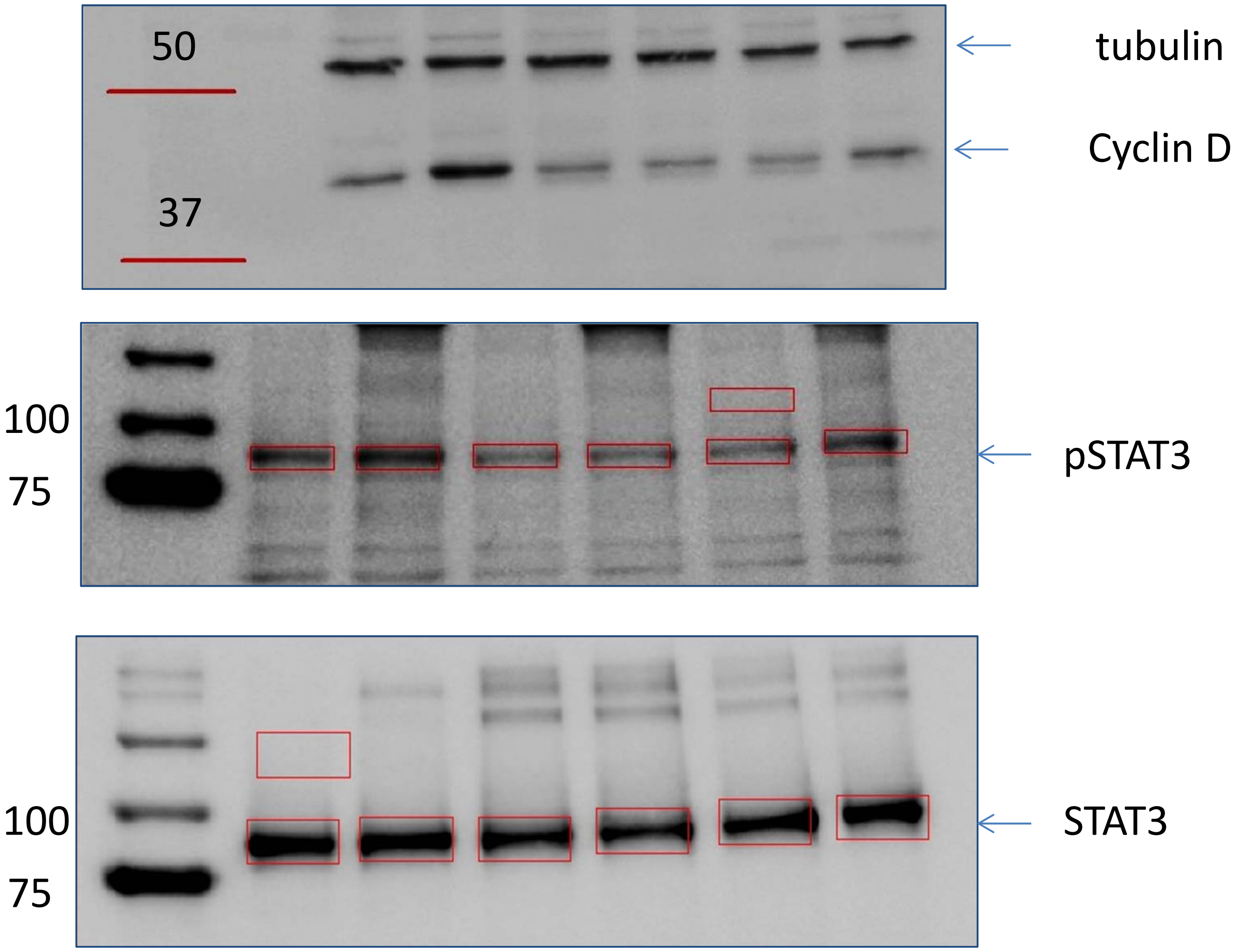

Representative Blots for Figure 6
